# Validation of a novel Multi-Gas sensor for volcanic HCl alongside H_2_S and SO_2_ at Mt. Etna

**DOI:** 10.1007/s00445-017-1114-z

**Published:** 2017-04-17

**Authors:** T. J. Roberts, T. Lurton, G. Giudice, M. Liuzzo, A. Aiuppa, M. Coltelli, D. Vignelles, G. Salerno, B. Couté, M. Chartier, R. Baron, J. R. Saffell, B. Scaillet

**Affiliations:** 1 Institut Pierre Simon Laplace, CNRS/UPMC, 4 place Jussieu, 75252 Paris, France; 2 Istituto Nazionale di Geofisica e Vulcanologia, sezione di Palermo, Via La Malfa 153, 90146 Palermo, Italy; 3 Dipartimento DiSTeM, Università di Palermo, Via Archirafi 36, 90123 Palermo, Italy; 4 Istituto Nazionale di Geofisica e Vulcanologia, Osservatorio Etneo, Piazza Roma 2, 95125 Catania, Italy; 5 Alphasense Ltd, Sensor Technology House, 300 Avenue West, Skyline 120, Great Notley, Braintree, Essex, CM77 7AA UK; 6 ISTO, CNRS/Université d’Orléans/BRGM, UMR 7327, 1a rue de la Férollerie, 45071 Orléans, France

**Keywords:** Multi-Gas instrument, Electronic nose, E-nose, Chlorine, Halogen, Volcanic outgassing, Open-system volcanic degassing

## Abstract

Volcanic gas emission measurements inform predictions of hazard and atmospheric impacts. For these measurements, Multi-Gas sensors provide low-cost in situ monitoring of gas composition but to date have lacked the ability to detect halogens. Here, two Multi-Gas instruments characterized passive outgassing emissions from Mt. Etna’s (Italy) three summit craters, Voragine (VOR), North-east Crater (NEC) and Bocca Nuova (BN) on 2 October 2013. Signal processing (Sensor Response Model, SRM) approaches are used to analyse H_2_S/SO_2_ and HCl/SO_2_ ratios. A new ability to monitor volcanic HCl using miniature electrochemical sensors is here demonstrated. A “direct-exposure” Multi-Gas instrument contained SO_2_, H_2_S and HCl sensors, whose sensitivities, cross-sensitivities and response times were characterized by laboratory calibration. SRM analysis of the field data yields H_2_S/SO_2_ and HCl/SO_2_ molar ratios, finding H_2_S/SO_2_ = 0.02 (0.01–0.03), with distinct HCl/SO_2_ for the VOR, NEC and BN crater emissions of 0.41 (0.38–0.43), 0.58 (0.54–0.60) and 0.20 (0.17–0.33). A second Multi-Gas instrument provided CO_2_/SO_2_ and H_2_O/SO_2_ and enabled cross-comparison of SO_2_. The Multi-Gas-measured SO_2_-HCl-H_2_S-CO_2_-H_2_O compositions provide insights into volcanic outgassing. H_2_S/SO_2_ ratios indicate gas equilibration at slightly below magmatic temperatures, assuming that the magmatic redox state is preserved. Low SO_2_/HCl alongside low CO_2_/SO_2_ indicates a partially outgassed magma source. We highlight the potential for low-cost HCl sensing of H_2_S-poor HCl-rich volcanic emissions elsewhere. Further tests are needed for H_2_S-rich plumes and for long-term monitoring. Our study brings two new advances to volcano hazard monitoring: real-time in situ measurement of HCl and improved Multi-Gas SRM measurements of gas ratios.

**Electronic supplementary material:**

The online version of this article (doi:10.1007/s00445-017-1114-z) contains supplementary material, which is available to authorized users.

## Introduction

Monitoring of volcanic gas emissions provides insight into subsurface degassing and outgassing processes with the aim of improved prediction of volcanic activity and hazards (e.g. Aiuppa et al. 2007a; Edmonds 2008), and provides the source data needed to develop atmospheric models of plume impacts (e.g. von Glasow 2010; Roberts et al. 2014b). The emitted gases include H_2_O, CO_2_, SO_2_, HCl, HF, H_2_S, CO, H_2_, HBr, HI and Hg in typical descending order of abundance, e.g. Fischer (2008), although emissions vary depending on magmatic state. Explosive eruptions account for about ~60% of global volcanic emissions (Halmer et al. 2002). Passively (quiescently), outgassing volcanoes are responsible for ~40%. Their mid-tropospheric plumes are difficult to detect by satellite and require day-to-day monitoring by ground-based instruments. Over the last decade, small in situ gas sensor instruments (Multi-Gas instruments) have been developed, enabling real-time measurements of emission composition at the volcano summit (Aiuppa et al. 2005b; Shinohara 2005). The low cost of Multi-Gas sensors and their ability to be automated make them a highly valuable technology for continuous monitoring of volcano H_2_O-CO_2_-SO_2_-H_2_S emissions. However, Multi-Gas instruments currently lack the ability to detect volcanic halogens despite their known importance as indicators of magmatic processes, and for atmospheric chemistry and deposition impacts. A further issue is measurement accuracy: Roberts et al. (2014a) showed that uncertainties and bias can arise in Multi-Gas-measured volcanic gas-ratios (e.g. H_2_S/SO_2_) even when well-calibrated. This is due to the non-instantaneous response times of the sensors. This study demonstrates a new capacity for in situ HCl monitoring by low-cost electrochemical sensors, demonstrated at quiescently outgassing Mt. Etna volcano, Italy, with improved accuracy of H_2_S/SO_2_ and HCl/SO_2_ gas ratios achieved by signal processing methods.

### Volcanic gas measurements and Multi-Gas

To quantify volcanic emissions requires both the gas flux and emission composition to be characterized. Volcanic SO_2_ fluxes are provided by remote sensing e.g. UV spectroscopy and previously COSPEC (e.g. Galle et al. 2002; Williams-Jones et al. 2008), or UV-camera-based spectroscopy (e.g. Mori and Burton 2006), and recently, IR-camera in the infra-red (Lopez et al. 2015). At several volcanoes including Mt. Etna, automated versions of these instruments have been installed to provide continuous (daytime, day-to-day) monitoring (Salerno et al. 2009). The emission composition (SO_2_ and other gases e.g. H_2_S, HCl) can be further characterized by in situ methods (e.g. gas/aerosol sampling onto filters) or remote sensing (e.g. Fourier transform infra-red spectroscopy (FTIR)), with data analysed to yield gas ratios relative to SO_2_. Combining these gas ratios with the SO_2_ gas flux thereby provides a comprehensive emission flux.

To determine the composition of volcanic emissions, in situ time-averaged sampling has been performed for many decades, using Giggenbach bottle traps and alkaline filter-packs, e.g. Aiuppa et al. (2005a), Shinohara and Witter (2005), Wittmer et al. (2014) and references therein. Such techniques can provide accurate HCl/SO_2_ ratios in the summit emissions and in sustained grounding downwind plume. However, deployment requires hazardous visits to the volcano summit followed by further costs in time-intensive laboratory analysis. Also, infrequent campaign-based monitoring might miss some composition changes. Remote sensing of HCl (e.g. by active FTIR with IR source) at individual summit craters is similarly typically limited to occasional field campaigns (La Spina et al. 2010). An automated FTIR instrument has been deployed at Stromboli summit, La Spina et al. (2013), using the hot crater vents and/or explosive activity as an IR source, but is impractical at Mt. Etna. Instead, weekly (daytime, weather dependent) FTIR monitoring in solar occultation mode measures the bulk plume composition but not individual crater emissions (e.g. Burton et al. 2003).

In this context, Multi-Gas instruments containing small sensors (Shinohara 2005; Shinohara and Witter 2005; Aiuppa et al. 2005b; Roberts et al. 2012) offer the capability for real-time in situ monitoring of several volcanic gases (typically SO_2_ and H_2_S by electrochemical sensor, CO_2_ and H_2_O by infra-red sensor), including long-term installations with data telemetry, e.g. at Italian volcanoes Mt. Etna and Stromboli, Aiuppa et al. (2007a, 2009) and Calvari et al. (2014). Specific campaigns have also deployed other portable in situ instruments alongside Multi-Gas to detect mercury (e.g. Witt et al. 2008) and ozone (Surl et al. 2015). Recent instrument advancements have widened the Multi-Gas small sensor approach to include H_2_ and CO (Aiuppa et al. 2011; Shinohara et al. 2011; Roberts et al. 2012; Moussallam et al. 2012). However, due to lack of HCl sensors suitable for Multi-Gas (except for a prototype study by Roberts et al. 2012), HCl detection is restricted to filter-pack sampling or by FTIR remote sensing. This approach of co-deploying filter-packs/FTIR alongside Multi-Gas (e.g. Shinohara and Witter 2005) determines a more comprehensive emissions composition on a campaign basis (or more regularly at volcanoes with nearby observatory facilities), but does not provide possibility for continuous in situ monitoring of HCl.

### Volcanic HCl emissions

Measuring HCl (alongside CO_2_ and SO_2_) in the volcanic emission is of strong interest. HCl outgasses at shallower depths than SO_2_ and CO_2_, thus HCl/SO_2_ can be an informative indicator of magma state and potentially might be used to improve monitoring and prediction of volcanic eruption hazards: Studies report both increasing and decreasing trends in HCl/SO_2_ related to volcanic activity, for example S/Cl mass ratios rising from 5 to 25 prior to an eruption event at Asama volcano (Noguchi and Kamiya 1963) and SO_2_/HCl molar ratios decreasing from 2.3 to 0.1 (Aiuppa et al. 2002) and varying between 0.1 and 7.1 (Aiuppa et al. 2004) during eruptive activity at Mt. Etna. Ohno et al. (2013) and references therein report HCl/SO_2_ decreasing from 0.6 to <0.1 following eruption at Mt. Aso and also highlight the role of hydrothermal and surface lake processes.

At Mt. Etna specifically, presence of multiple craters with distinct emissions adds a further complexity. Multi-Gas has been used to trace CO_2_/SO_2_ emissions during and following an eruption event, Aiuppa et al. (2006). However, temporal variations in emitted SO_2_/HCl and CO_2_/SO_2_ ratios at the crater sites are observed even without overall change in magma supply. For example, episodic rise of deeply outgassed CO_2_- and H_2_-rich bubbles has been proposed to explain temporal variations in Multi-Gas CO_2_/SO_2_ and H_2_/SO_2_ at Mt. Etna (Shinohara et al. 2008; Aiuppa et al. 2011), whilst La Spina et al. (2010) proposed a branched conduit model to explain CO_2_-SO_2_-HCl variations. A wide compositional range in molar SO_2_/HCl is reported (0.1–14.7), Aiuppa (2009) and references therein, that is wider and typically shifted to lower gas ratios (except during fire fountain events) than expected from closed system degassing, 3.7–9.7, Spilliaert et al. (2006a, 2006b). This indicates efficient separation of gas and melt within the plumbing system. Varying degrees of SO_2_ and halogen outgassing during magma ascent can be invoked to explain the range in reported surface-measured emissions of SO_2_/HCl (see Aiuppa 2009). There is also some melt-inclusion evidence at Mt. Etna for Cl enhancement in the melt at low pressures (Spilliaert et al. 2006b). Clearly, a more frequent measurement of volcanic HCl at each of the craters could further understanding of the degassing and outgassing behaviour.

Volcanic emission monitoring also provides input to atmospheric models. Impacts from halogens include ecosystem damage from not only acid deposition (Delmelle 2003) but also plume reactive halogen (BrO, OClO) chemistry that destroys tropospheric ozone, converts NO_x_ into HNO_3_ (Roberts et al. 2009) and may enhance deposition of mercury (von Glasow 2010). Recent model studies highlight potential impacts of volcanic halogens on stratospheric ozone, either by direct eruptive injection (e.g. Cadoux et al. 2015) or by passive outgassing combined with convective processes (Jourdain et al. 2015). Mather (2015) reviews the environmental importance of volcanic emissions emphasizing halogens but highlights uncertainties in their emissions and plume processing.

### Sensor response time as a source of error in Multi-Gas gas ratios

A source of error in gas ratios from Multi-Gas arises from differing sensor response times, which depend on both sensor and gas properties. “Standard” analysis of Multi-Gas data (see “Standard analysis of Multi-Gas SO_2_ and H_2_S”) implicitly assumes instantaneous or identical sensor response times. Through forward modelling of sensor response, Roberts et al. (2014a) showed that this assumption can cause measurement errors and bias in the gas ratio, especially under rapidly fluctuating gas exposure e.g. at the crater-rim where plume exposure depends on local wind-fields. Errors are magnified by the subtraction of interferences (of SO_2_ on the H_2_S measurement) in data post-processing. At Mijake-jima Volcano, this led to 30–50% errors in standard analysis of Multi-Gas H_2_S/SO_2_ measured at the crater-rim, but not in the sustained (and more slowly fluctuating) downwind plume. This systematic error is independent of any calibration errors and is particularly large at low H_2_S/SO_2_. As consequence, Multi-Gas H_2_S/SO_2_ ratios are rarely reported from H_2_S-poor volcanoes.

Data integration can partially compensate for this source of error but only for individual (non-overlapping) gas pulse events, Roberts et al. (2014a). Here, systems engineering signal processing methods are applied in combination with laboratory sensor characterisations to deliver an improved analysis of H_2_S/SO_2_ and HCl/SO_2_ gas ratios.

## Multi-Gas sensor theory

### Standard analysis of Multi-Gas SO_2_ and H_2_S

Standard analysis of Multi-Gas data implicitly assumes an instantaneous sensor response. The SO_2_ gas abundance, [SO_2_(*t*)] in ppmv, is determined by Eq. 1, where Signal_SO2-AE_(*t*) is the signal of the SO2-AE sensor (with any baseline removed), whose sensitivity, *sens*_SO2_, is determined by calibration. Typically, *sens*_SO2_ is in nA/ppmv. The sensor Signal_SO2-AE_(*t*) is in nA, which is converted to a voltage and recorded by the Multi-Gas.

1 $$ \left[{SO}_2(t)\right]=\frac{Signal_{SO2- AE}(t)}{sens_{SO2}} $$

Multi-Gas H_2_S sensors such as H2S-AE exhibit cross-sensitivity to SO_2_ as well as sensitivity to H_2_S. This interference is subtracted from the sensor signal in data post-processing, following Eq. 2 where sens_H2S_ is the sensitivity to H_2_S and xsens_SO2_ is the cross-sensitivity to SO_2_, [SO_2_(*t*)], which is provided by 1.

2 $$ \left[{H}_2 S(t)\right]=\frac{Signal_{H2 S- AE}(t)-{xsens}_{SO2}\cdot \left[{SO}_2(t)\right]}{sens_{H2 S}} $$

Linear regression on a scatter plot of [H_2_S(*t*)] versus [SO_2_(*t*)] determines the gas ratio. However, this ratio is prone to biases if sensors have non-identical response times, see “Introduction”. A similar analysis of HCl/SO_2_ by equivalent equations to Eqs. 1 and 2 (removing the H_2_S interference from the HCl-A1 signal) could also incur similar errors. This study attempts to reduce such biases in Multi-Gas gas ratios by modelling the sensor response.

### SRM

Numerical models can be used to simulate the sensor’s transient response, based on signal processing methods from systems engineering (e.g. Ljung 1987) assuming a linear, time-invariant, causal model, fitted to sensor calibration data (typically responding to a step-change or gas pulse). Below, the forward sensor response model (SRM) is described, whose parameters are quantified from batch calibrations in “Sensor characterization: sensitivity, cross-sensitivities, T90 and SRM”. Approaches to use SRM in field-data analysis to derive gas ratios are developed in ‘Results’.

The rise/decay response curve of an electrochemical sensor responding to a step-change in gas abundance is broadly exponential, i.e. follows Eq. 3 where Signal is the sensor signal over time *t*, responding (in proportion to its sensitivity, *sens*) to a step-change in target gas abundance from [*X*_start_] to [*X*_final_]. The time constant parameter, *τ*, is the time to reach 1/e of the signal change. The response time to reach 90% of the signal change, T90, is related to *τ* by T90 = Log_e_[10] *τ*. Typically, *T*_90_ and *τ* are independent of the gas abundance change, but see “Sensor characterization: sensitivity, cross-sensitivities, T90 and SRM” for further discussion.

3 $$ {Signal}_{sens or}^{sens}(t)=\left[{X}_{final}\right]\cdot sens+\left(\left[{X}_{start}\right]-\left[{X}_{final}\right]\right)\cdot sens\cdot Exp\left[-\frac{t}{\tau}\right] $$

Equation 3 can be rearranged as follows. Writing the signal at time *t*-Δ*t* as Eq. 4, then multiplying Eq. 4 by *F* = Exp[−Δ*t*/*τ*] and both adding and subtracting [*X*_final_]∙sens from the right-hand-side yields Eq. 5.

4 $$ {Signal}_{sens or}^{sens}\left( t-\Delta t\right)=\left[{X}_{final}\right]\cdot sens+\left(\left[{X}_{start}\right]-\left[{X}_{final}\right]\right)\cdot sens\cdot Exp\left[-\frac{t}{\tau}\right]\cdot Exp\left[\frac{\Delta t}{\tau}\right] $$

5 $$ F\cdot {Signal}_{sens or}^{sens}\left( t-\Delta t\right)=\left[{X}_{final}\right]\cdot sens\cdot \left( F-1\right)+\left[{X}_{final}\right]\cdot sens+\left(\left[{X}_{start}\right]-\left[{X}_{final}\right]\right)\cdot sens\cdot Exp\left[-\frac{t}{\tau}\right] $$

Finally, substituting Eq. 3 and rearranging yields Eq. 6 that describes the Signal(*t*), as a function of [*X*_final_] and *sens* and the previously recorded signal, Signal(*t*-Δ*t*).

6 $$ {Signal}_{sens or}^{sens}(t)={Signal}_{sens or}^{sens}\left( t-\Delta t\right)\cdot F+\left[{X}_{final}\right]\cdot sens\cdot \left(1- F\right) $$

Parameter *F* (0 ≤ *F* ≤ 1) describes the amount of decay between successive samples and is related to the time-constant T90 or *τ*, in seconds, by Eq. 7, where Δ*t* is the sampling period in seconds.

7 $$ F= EXP\left(\frac{Log_e\left[0.1\right]\cdot \Delta t}{T_{90}}\right)= EXP\left(-\frac{\Delta t}{\tau}\right) $$

“Sensor characterization: sensitivity, cross-sensitivities, T90 and SRM” section finds the signal is slightly over-damped compared to Eq. 6, which we represent using a second-order SRM involving Signal(*t*-2Δ*t*) terms. Mathematically, this is equivalent to the weighted addition of two first-order SRMs (labelled A and B), Eqs. 8–10, where *W* is a weighting factor (between 0 and 1).

8 $$ {Signal}_{sensor}^{sensA}(t)={Signal}_{sensor}^{sensA}\left( t-\Delta t\right)\cdot {F}_A+\left[ X(t)\right]\cdot sens\cdot \left(1-{F}_A\right) $$

9 $$ {Signal}_{sensor}^{sensB}(t)={Signal}_{sensor}^{sensB}\left( t-\Delta t\right)\cdot {F}_B+\left[ X(t)\right]\cdot sens\cdot \left(1-{F}_B\right) $$

10 $$ {Signal}_{sens or}^{sens}(t)= W\cdot {Signal}_{sens or}^{sens A}\left( t-\Delta t\right)+\left(1- W\right)\cdot {Signal}_{sens or}^{sens B}(t) $$

If [*X*(*t*)] is an interference rather than target gas of the sensor, the sensitivity, *sens*, is replaced by the cross-sensitivity, *xsens*, in Eqs. 3–10. The overall sensor signal is the sum of the signal response to the sensitivity (target) gas and any interference gases, Eq. 11.

11 $$ {Signal}_{Sensor}^{simulated}(t)={Signal}_{Sensor}^{sens}(t)+{Signal}_{Sensor}^{\operatorname{int}\mathit{\operatorname{erf}}}(t) $$

## Methods

### Terminology

We refer to gas abundance in parts per million by volume (ppmv). This is equivalent to a mixing ratio in μmol/mol. Concentration refers to molecules per unit volume of air. Gas ratio is the ratio of two measured gas abundances, i.e. a molar ratio.

### Direct exposure Multi-Gas instrument and sensors

**Fig. 1:**
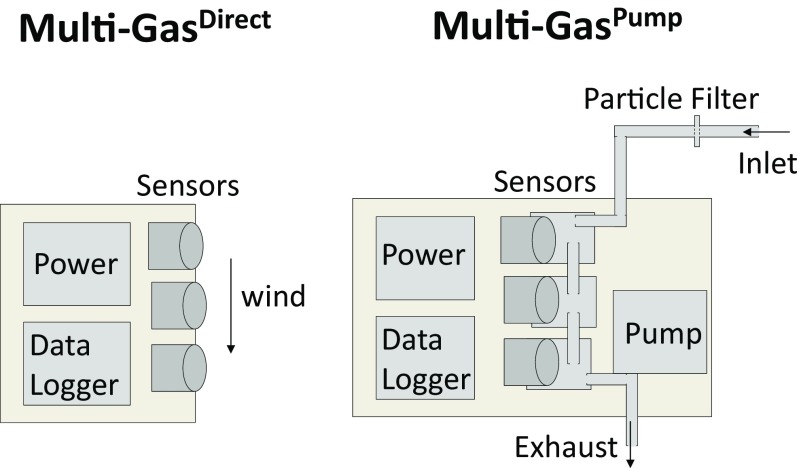
Schematics of “direct exposure” instrument, Multi-Gas^Direct^, where the sensors are directly exposed to the atmosphere, and Multi-Gas^Pump^, where gases are pumped through the instrument

A “direct exposure” Multi-Gas instrument, Multi-Gas^Direct^, was developed that operates without a pump. Instead, the electrochemical sensors (SO2-AE, SO2-A4, H2S-AE, HCl-A1 manufactured by Alphasense Ltd.; sensor names as per Alphasense.com) were exposed directly (simultaneously) to the ambient air, Fig. 1. Advantages include lighter weight (reduced to few 100 g) and lower power consumption (equivalent to 4 AA batteries for 1–2 weeks) than a pumped Multi-Gas. The instrument used low-noise electronics (3 mV peak-to-peak) with the sensor output (0 to 2.5 V) logged at 0.1 Hz using HOBO U12–006 data logger (accuracy ±2 mV ± 2.5% of absolute reading, precision 0.6 mV). Temperature next to the sensors (close to ambient given no instrument heating) was monitored using a PT1000 resistance thermometer. Electrochemical sensor sensitivities are temperature-dependent, but at the ambient field-temperatures encountered (10–15 °C), the sensitivity is within 3% of the calibrations (at 20 °C). Sensor specifications report rms sensor noise <1 ppmv for HCl-A1, <1.5 ppmv for SO2-AE, <0.5 ppmv for H2S-AE and report 15 ppbv for SO2-A4 (±2 standard deviations). Thus, SO2-A4 has a higher sensitivity than SO2-AE and yields better resolution data but exhibits a lower range (~6 ppmv) compared to SO2-AE (~38 ppmv), for the electronics board used. For the highly polluted crater-rim observations of this study, the SO_2_ analysis focuses on SO2-AE. The electrochemical sensor signal depends on diffusion rates so is proportional to ppmv abundance (not concentration) and required no pressure correction. The datasheet sensor pressure range is 800–1200 hPa (15–90% RH), but there are no known sensor issues at ~700 hPa (typical pressure at Mt. Etna summit, 3.3 km asl), Alphasense, pers. com. A second instrument, Multi-Gas^Pump^ (of standard design with pump), containing an additional electrochemical sensor for SO_2_ (3ST/F) as well as sensors for CO_2_, H_2_O, was co-deployed. Details are in Supplementary Material and Pering et al. (2014).

### Sensor characterization: sensitivity, cross-sensitivities, T90 and SRM

Room-temperature individual sensor-specific calibrations prior to the field-campaign found the following sensitivities to target gases for SO2-AE, SO2-A4, H2S-AE and HCl-A1: 72, 438, 88 and 113 nA/ppmv (where 1 nA is converted to 0.8 mV by the electronics board), respectively, determined from the sensor signal rise during 10 min gas exposure. Batch calibrations (of groups of sensors) also quantified generic cross-sensitivity of H2S-AE to SO_2_ (~14 ± 0.5%, with one outlier at 9.3%), typical for Multi-Gas H_2_S sensors, Table 1.

**Table 1:** Sensor sensitivities, cross-sensitivities and T90 sensor response times determined by laboratory calibration and used in the sensor response model (SRM) analysis of H_2_S/SO_2_ and HCl/SO_2_

Sensor	SO_2_-AE	H2S-AE	H2S-AE	HCl-A1	HCl-A1
Gas	SO_2_	H_2_S	SO_2_	HCl	H_2_S
Sensitivity (nA/ppmv)	72	88	–	113	–
Cross-sensitivity (% of sensitivity)	–	–	14 (13.5–14.5)	–	210 (170–250)
T90 (s)	13 (10–15)	25 (20–50)	50 (40–70)	150 (100–250)	250 (200–300)
Second-order SRM asc (desc)					
T90 (s) for parameter F^A^	–	14	35	40 (20)	65 (25)
T90 (s) for parameter F^B^	–	180	300	300 (200)	500 (500)
Weighting W	–	0.86	0.93	0.66 (0.66)	0.66 (0.8)

Sensitivities are for the specific sensors used in this study. Cross-sensitivity range (in brackets) reflects calibrations on batches of sensors of the same type. Sensor T90’s (and range) were determined from batch calibrations. Both ascending and descending (in brackets) SRM parameters are given for HCl-A1. See “Sensor characterization: sensitivity, cross-sensitivities, T90 and SRM” section for details

**Fig. 2:**
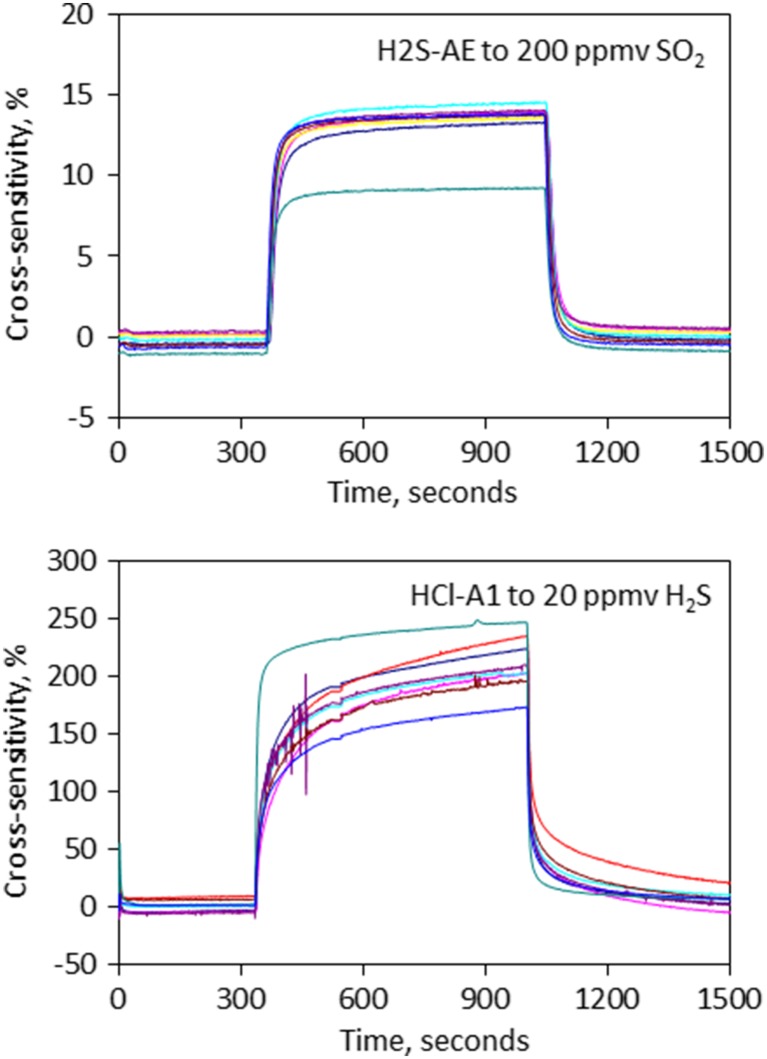
Cross-sensitivity of H2S-AE to SO_2_ and HCl-A1 to H_2_S determined from laboratory calibrations on batches of sensors. Units are percentage of sensor sensitivity to target gas. H2S-AE exhibits a cross-sensitivity of ~14 ± 0.5% (with one outlier at 9.3%) to SO_2_, and HCl-A1 exhibits a 170–250% (mean 210%) cross-sensitivity to H_2_S

HCl-A1 sensor interferences have not been characterized previously. HCl-A1 exhibits negligible cross-sensitivities to major volcanic gases SO_2_ or CO_2_, Fig. S1, and no strong evidence of HF cross-sensitivity. The 10 min calibrations do identify interferences from HBr and H_2_S equivalent to cross-sensitivities of ~50 and 170–250% (mean 210%), respectively, Fig. 2. The impact of HBr on the HCl measurement is expected to be negligible in volcanic plumes given HCl/HBr ~ 10^3^, with molar Cl/Br ratio for Mt. Etna quantified by Wittmer et al. (2014) as 500–700 in 2010–2012. Whilst the cross-sensitivity to H_2_S is large, the H_2_S interference on the HCl measurement is expected to be small (but not insignificant) in Mt. Etna’s HCl-rich, H_2_S-poor plume.

Laboratory calibrations show slight variations in HCl-A1 sensor output of 10% (2σ) sensitivity and 2 ppmv (2σ) baseline following initial exposure Fig. S2. Reported long-term stability over 100 days (13 calibrations) for HCl-B1 (a larger version of sensor HCl-A1) is 17% (2σ) for sensitivity and 2 ppmv (2σ) for baseline, Alphasense pers. com. For comparison, the sensitivity stability for SO2-AE is reported as <4% drift per year.

**Fig. 3:**
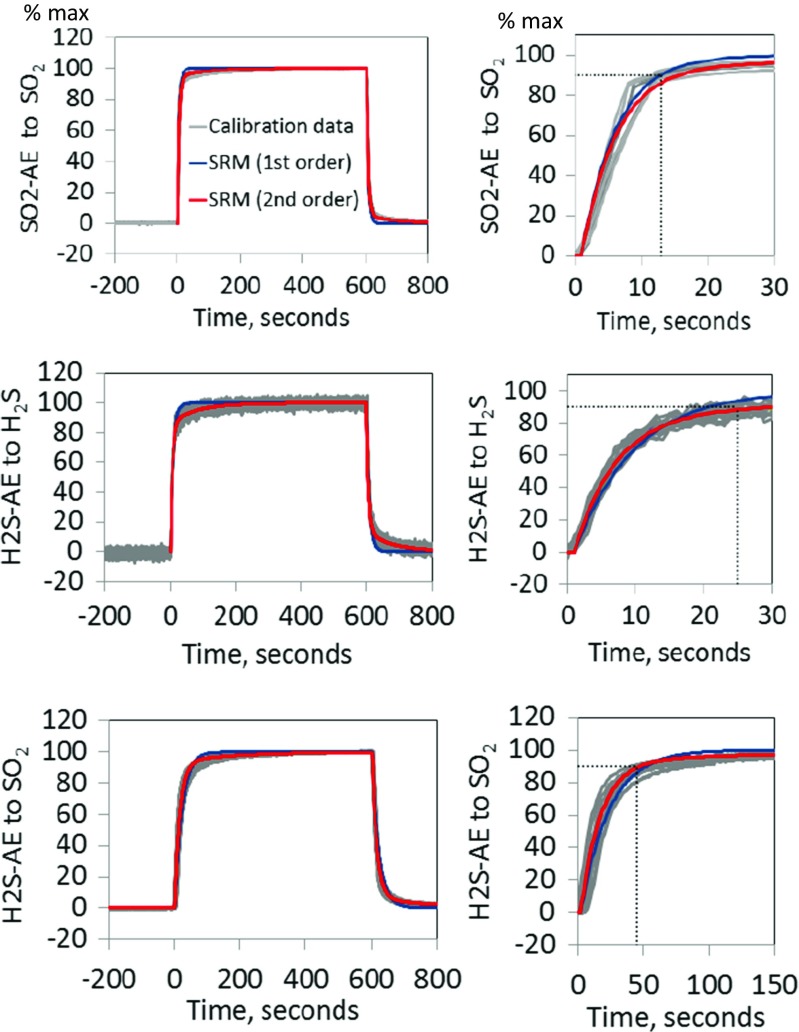
Laboratory calibrations (in batches of same sensor type) quantifying SO2-AE and H2S-AE sensor response to and following a 10 min gas exposure (between 0 and 600 s). The gas abundances used were 400 ppmv SO_2_, 20 ppmv H_2_S and 200 ppmv SO_2_, respectively. Sensor signals have been normalized to reach 100% at the end of the 10 min exposure. Sensor response models (SRM) of first- (*blue*) and second-order (*red*) are fitted to the sensor signal. The sensor T90 is also shown (*dotted black lines*), Table 1. Sensor response to and recovery from the gas pulse are similar

**Fig. 4:**
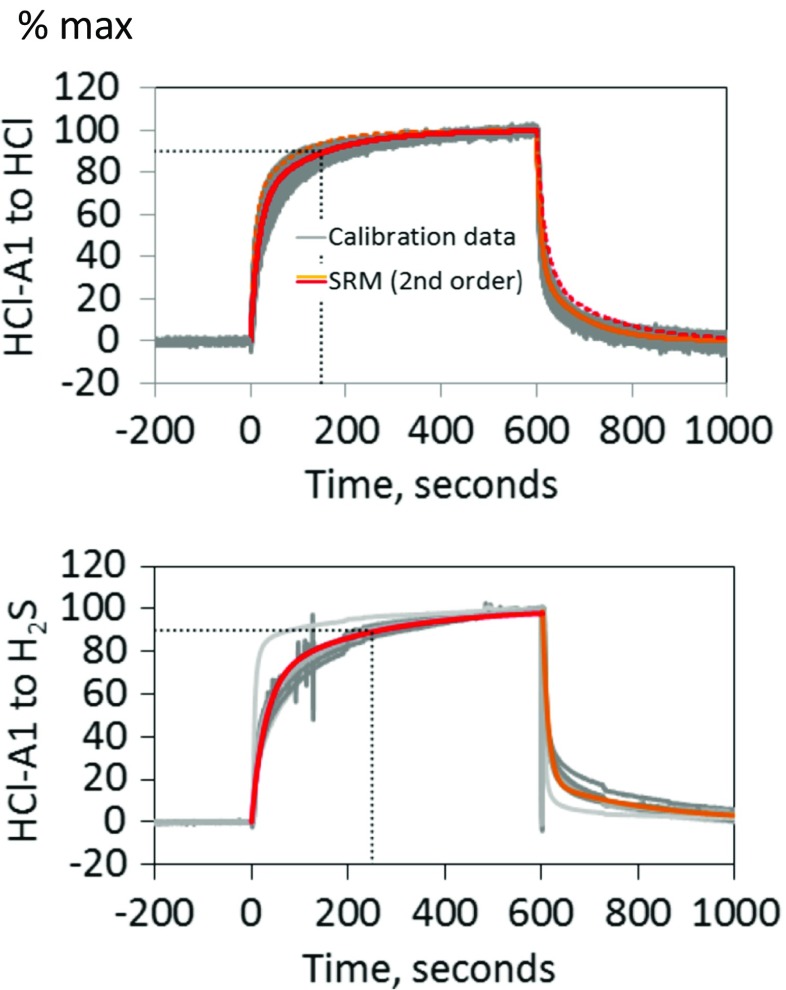
Laboratory calibrations (in batches of the same sensor type) quantifying HCl-A1 sensor response to and following a 10 min gas exposure (between 0 and 600 s). The gas abundances used were 25 ppmv HCl and 20 ppmv H_2_S. Sensor signals have been normalized to reach 100% at the end of the 10 min exposure. Sensor response models (SRM) are also shown, where the response to the gas pulse (*red*) is somewhat slower than recovery following the gas pulse (*orange*); see SRM parameters in Table 1. The sensor T90 is also shown (*dotted black lines*)

Batch calibrations (10 min exposure) were used to characterize sensor response times, finding T90’s of ~12 s, 20–50 s and 100–250 s, respectively, for SO2-AE, H2S-AE and HCl-A1, Figs. 3 and 4, Table 1. Sensor response to the gas pulse is non-instantaneous and can be traced and quantified by the fitted SRM’s. Typically, a slightly better agreement for the second-order SRM (red) than the first-order SRM (blue) indicates that the signal is over-damped. For HCl-A1 (but not SO2-AE, H2S-AE), the descent response is slightly faster than the ascent. Also, the HCl-A1 response exceeds the 10 min experiment duration: Tests over 1 h observe signals to HCl (H_2_S) up to 25% (7%) higher than at 10 min, Fig. S2. This may reflect auto-activation with some baseline drift. Whilst this would imply proportionally higher (cross-)sensitivity and slower response, these opposing effects largely cancel in SRM analysis of our field-data (‘Field measurements’), although might have greater importance for longer plume exposures.

## Results

### Field measurements

**Fig. 5:**
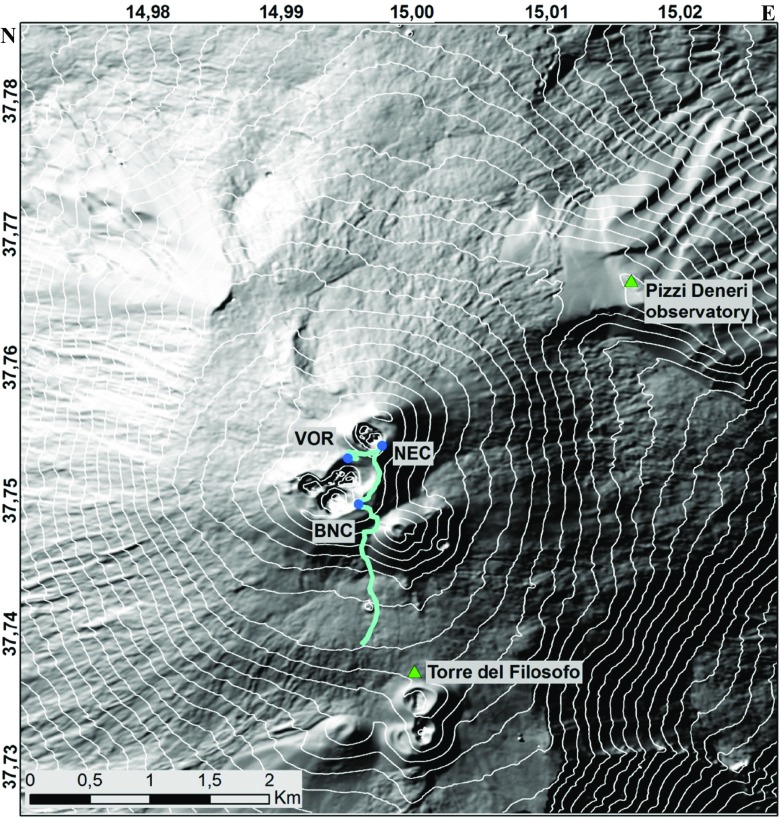
Map of Mt. Etna volcano summit showing locations of the Multi-Gas measurements made consecutively at VOR (Voragine), NEC (North-East Crater) and Bocca Nuova (BN) crater-rims, and the descent path from BN that sampled progressively more dilute grounding plume

Emissions from passively outgassing Mt. Etna on 2 October 2013 were detected at locations shown in Fig. 5. Summit measurements were made consecutively at the three active crater-rim sites: Voragine (VOR), North East Crater (NEC) and Bocca Nuova (BN). Strong north-westerly winds were observed, also confirmed by meteorological balloon soundings in Trapani that indicate 12 m s^−1^ (see http://weather.uwyo.edu/upperair/sounding.html). This allowed the plume to be traced for several hundred meters along the volcano flank during descent from BN.

**Fig. 6:**
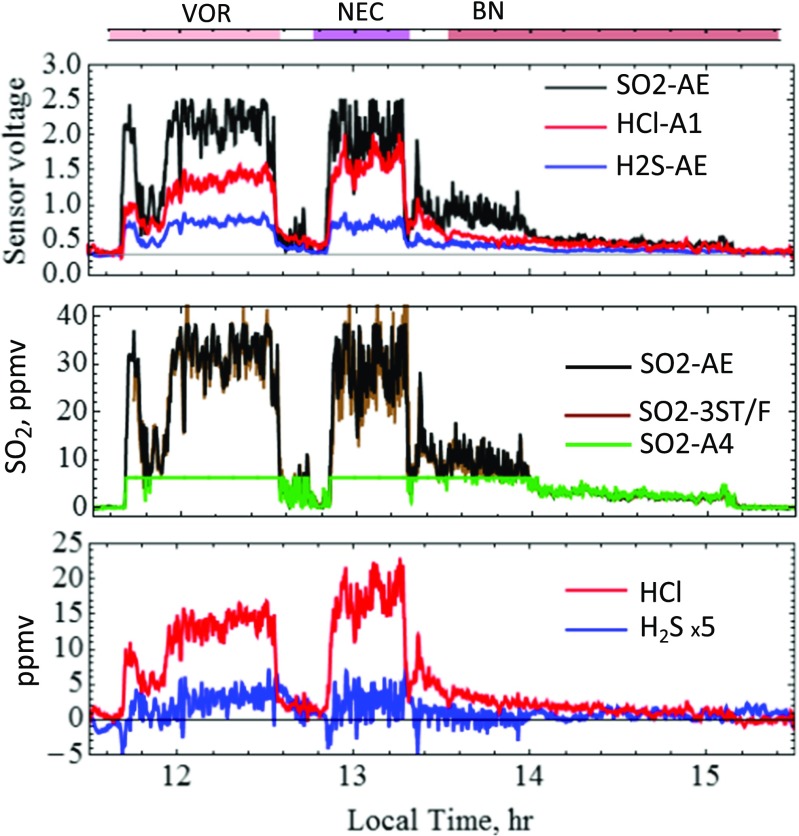
Multi-Gas^Direct^ SO2-AE, H2S-AE and HCl-A1 sensor signals with SO_2_, H_2_S and HCl gas abundances derived by standard data analysis. Noise in the H_2_S time series is primarily caused by sensor response effects, and the HCl time series shows evidence for slow sensor response relative to SO_2_. The SO_2_ time series derived from SO2-AE is shown alongside measurements by two other electrochemical sensors, SO2-A4 in Multi-Gas^Direct^ and SO2-3ST/F in Multi-Gas^Pump^. Time periods for analysis of VOR, NEC and BN gas ratios are indicated

To gain an overview, gas abundance time series were first derived by “standard analysis” (Eqs. 1 and 2) from the raw signals, Fig. 6. Crater-rim emissions are observed as elevated gas abundance over tens of minutes, interspersed with periods of relatively clean air (between craters). SO_2_ abundances reached up to ~35 ppmv at VOR and NEC but were somewhat lower at BN where more dilute plume was sampled. The visually slower response of HCl compared to SO_2_ and noise in the H_2_S time series underline the need to consider sensor response times in determining gas ratios. Representative VOR, NEC and BN periods used for further data analysis are indicated, where the BN period includes plume measurements both at the crater edge and 10s–100s of metres from the crater, and excludes the more dilute gas encountered between craters at ~13h15 LT. From ~14 LT onwards, successively more dilute grounding plume was sampled during descent southwards from BN.

**Fig. 7:**
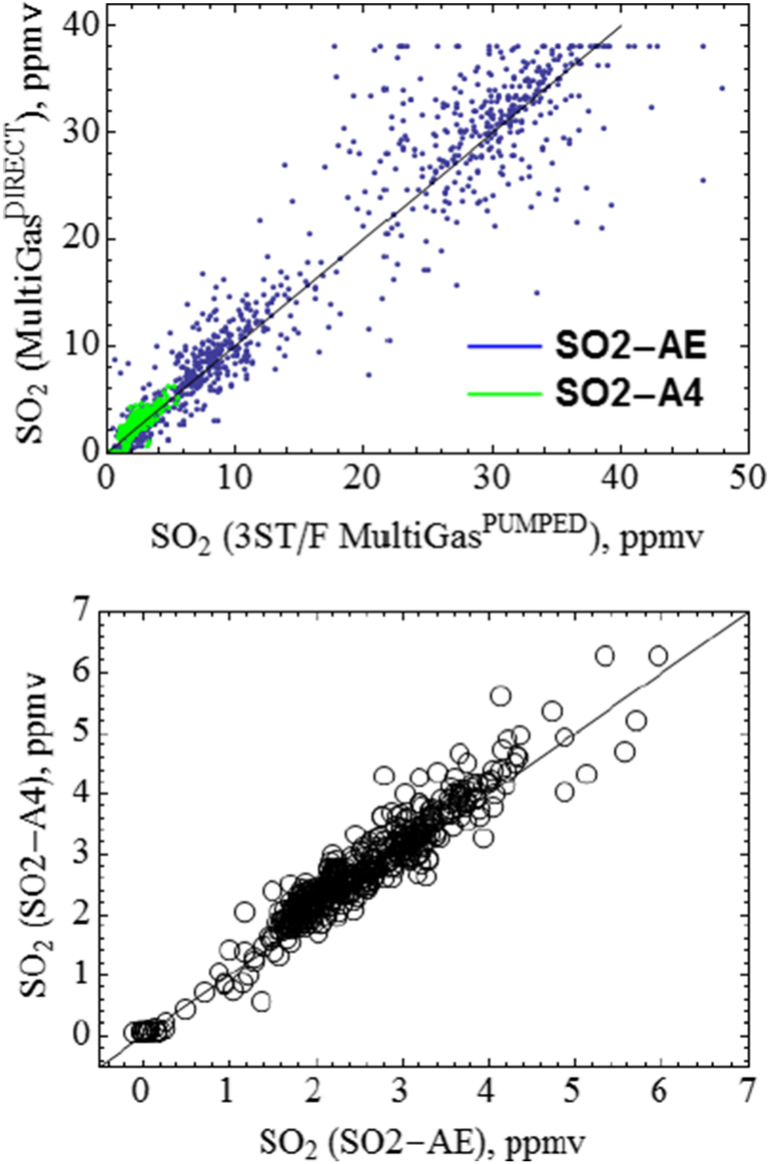
Direct comparison of SO_2_ co-measured by SO2-AE and SO2-A4 in Multi-Gas^Direct^, and SO2-3ST/F in Multi-Gas^Pump^. Linear regression yields 1 ± 0.05 with correlation coefficients >0.9 for the full-time series. The two Multi-Gas sensors were deployed within centimetre distance (exception: metres distance during descent from BN)

A point-by-point comparison finds good agreement in SO_2_ measured by SO2-AE and SO2-A4 in Multi-Gas^Direct^ and the 3ST/F SO_2_ sensor in Multi-Gas^Pump^, Fig. 7. Correlation coefficients are >0.9 over the whole time series, with scatter plot gradient 1 ± <0.05.

### Development of SRM data analysis approach for H_2_S/SO_2_ and HCl/SO_2_ gas ratios

**Fig. 8:**
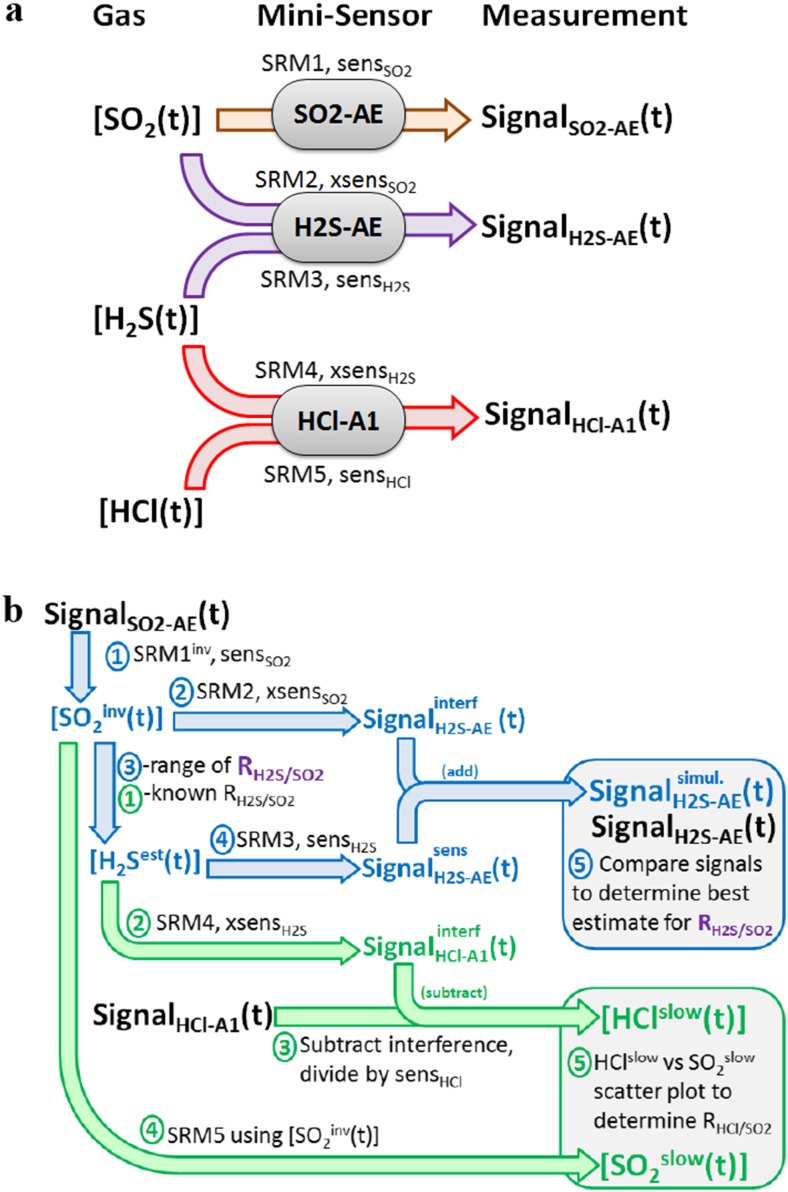
Flow charts illustrating sensor response to time-varying gas abundance and the analysis of sensor signals to yield H_2_S/SO_2_ and HCl/SO_2_ volcanic gas ratios. **a** Gases SO_2_, H_2_S and HCl induce signals in SO2-AE, H2S-AE and HCl-A1 sensors according to their sensitivity and cross-sensitivities (sens, xsens) and sensor response (SRM). **b** Analysis of SO2-AE, H2S-AE and HCl-A1 sensor signals to yield H_2_S/SO_2_ and HCl/SO_2_ gas ratios. The analysis considers sensitivities, cross-sensitivities and their SRM functions. For details, see “Development of SRM data analysis approach for H_2_S/SO_2_ and HCl/SO_2_ gas ratios” section

Figure 8a illustrates how the inputs (SO_2_, H_2_S, HCl gas abundances) to the sensors SO2-AE, H2S-AE and HCl-A1 yield three output signals, two of which are the sum of sensitivity and interference signals, i.e. involving five SRMs in total: labelled 1 to 5 for SO2-AE to SO_2_, H2S-AE to SO_2_, H2S-AE to H_2_S, HCl-A1 to H_2_S and HCl-A1 to HCl, with corresponding (cross)-sensitivities and response parameters. We propose two SRM approaches to analyse the observed sensor signals to determine molar gas ratios (*R*_H2S/SO2_, *R*_HCl/SO2_).

Firstly, for H_2_S/SO_2_, (1) inversion of the SO2-AE sensor signal yields an estimated yet noisy SO_2_ abundance [SO_2_(*t*)^inv^], Eq. 10. Here, a first-order SRM was used for this inversion, as the second-order SRM inversion proved too noisy.

10 $$ \left[{SO}_2^{inv}(t)\right]=\frac{Signal_{SO2- AE}(t)-{Signal}_{SO2- AE}\left( t-\Delta t\right)\cdot {F}_{SO2- AE}}{sens_{SO2}\cdot \left(1-{F}_{SO2- AE}\right)} $$

(2) [SO_2_^inv^(*t*)] is used with (forward modelled) SRM2 to simulate the interference from SO_2_ on H2S-AE, Eqs. 11–13.

11 $$ {Signal}_{H2 S- AE}^{\operatorname{int} erfA}(t)={Signal}_{H2 S- AE}^{\operatorname{int} erfA}\left( t-\Delta t\right)\cdot {F}_{H2 S- AE}^{\operatorname{int} erfA}+\left[{SO}_2^{inv}(t)\right]\cdot {xsens}_{H2 S- AE}\cdot \left(1-{F}_{H2 S- AE}^{\operatorname{int} erfA}\right) $$

12 $$ {Signal}_{H2 S- AE}^{\operatorname{int} erfB}(t)={Signal}_{H2 S- AE}^{\operatorname{int} erfB}\left( t-\Delta t\right)\cdot {F}_{H2 S- AE}^{\operatorname{int} erfB}+\left[{SO}_2^{inv}(t)\right]\cdot {xsens}_{H2 S- AE}\cdot \left(1-{F}_{H2 S- AE}^{\operatorname{int} erfB}\right) $$

13 $$ {Signal}_{H2 S- AE}^{\operatorname{int}\mathit{\operatorname{erf}}}(t)={W}_{H2 S- AE}^{\operatorname{int}\mathit{\operatorname{erf}}}\cdot {Signal}_{H2 S- AE}^{\operatorname{int} erfA}(t)+\left(1-{W}_{H2 S- AE}^{\operatorname{int}\mathit{\operatorname{erf}}}\right)\cdot {Signal}_{H2 S- AE}^{\operatorname{int} erfB}(t) $$

(3) The H_2_S abundance [H_2_S(*t*)^est^] is estimated by the product of [SO_2_(*t*)^inv^] with a range of possible *R*_H2S/SO2_, Eq. 14.

14 $$ \left[{H}_2{S}^{est}(t)\right]=\left[{{ S O}_2}^{inv}(t)\right]\cdot {R}_{H2 S/ SO2} $$

(4) [H_2_S^est^(*t*)] is used with SRM3 to simulate the sensitivity signal of H_2_S on H2S-AE, Eqs. 15–17.

15 $$ { S ignal}_{H2 S- AE}^{s ensA}(t)={ S ignal}_{H2 S- AE}^{s ensA}\left( t-\Delta t\right)\cdot {F}_{H2 S- AE}^{s ensA}+\left[{H}_2{S}^{est}(t)\right]\cdot {s}_{H2 S AE}\cdot \left(1-{F}_{H2 S- AE}^{s ensA}\right) $$

16 $$ { S ignal}_{H2 S- AE}^{s ensB}(t)={ S ignal}_{H2 S- AE}^{s ensB}\left( t-\Delta t\right)\cdot {F}_{H2 S- AE}^{s ensB}+\left[{H}_2{S}^{est}(t)\right]\cdot {s}_{H2 S- AE}\cdot \left(1-{F}_{H2 S- AE}^{s ensB}\right) $$

17 $$ {Signal}_{H2 S- AE}^{sens}(t)={W}_{H2 S- AE}^{sens}\cdot {Signal}_{H2 S- AE}^{sens A}(t)+\left(1-{W}_{H2 S- AE}^{sens}\right)\cdot {Signal}_{H2 S- AE}^{sens B}(t) $$

(5) Adding the two (sensitivity and interference) signals yields an overall simulated signal, Eq. 18.

18 $$ { S ignal}_{H2 S- AE}(t)={ S ignal}_{H2 S- AE}^{sens}(t)+{S}_{H2 S- AE}^{\operatorname{int}\mathit{\operatorname{erf}}}(t) $$

Finally, the simulated Signal_H2SAE_ is compared to the observed Signal_H2SAE_ for a range of *R*_H2S/SO2_. Best agreement signifies optimal choice of R_H2S/SO2_.

Secondly for HCl/SO_2_, (1) the H_2_S abundance is first estimated from the product of [SO_2_^inv^(*t*)] with *R*_H2S/SO2_ provided above. (2) The interference of H_2_S on the HCl-A1 signal is simulated by SRM4, Eqs. 19–21.

19 $$ { S ignal}_{H Cl- A1}^{\operatorname{int} erfA}(t)={ S ignal}_{H Cl- A1}^{\operatorname{int} erfA}\left( t-\Delta t\right)\cdot {F}_{H Cl- A1}^{\operatorname{int} erfA}+\left[{H}_2{S}^{est}(t)\right]\cdot {xsens}_{H2 S}\cdot \left(1-{F}_{H Cl- A1}^{\operatorname{int} erfA}\right) $$

20 $$ { S ignal}_{H Cl- A1}^{\operatorname{int} erfB}(t)={ S ignal}_{H Cl- A1}^{\operatorname{int} erfB}\left( t-\Delta t\right)\cdot {F}_{H Cl- A1}^{\operatorname{int} erfB}+\left[{H}_2{S}^{est}(t)\right]\cdot {xsens}_{H2 S}\cdot \left(1-{F}_{H2 S- A1}^{\operatorname{int} erfB}\right) $$

21 $$ {Signal}_{HCl- A1}^{\operatorname{int}\mathit{\operatorname{erf}}}(t)={W}_{HCl- A1}^{\operatorname{int}\mathit{\operatorname{erf}}}\cdot {Signal}_{HCl- A1}^{\operatorname{int} erfA}(t)+\left(1-{W}_{HCl- A1}^{\operatorname{int}\mathit{\operatorname{erf}}}\right)\cdot {Signal}_{HCl- A1}^{\operatorname{int} erfB}(t) $$

(3) This interference signal is subtracted from the observed Signal_HCl-A1_ to yield the sensitivity signal of HCl-A1, Eq. 22. The sensitivity signal is divided by the sensitivity to yield a slow response HCl abundance [HCl^slow^(*t*)], Eq. 23.

22 $$ {Signal}_{HCl- A1}^{sens}={Signal}_{HCl- A1}-{Signal}_{HCl- A1}^{\operatorname{int}\mathit{\operatorname{erf}}} $$

23 $$ \left[{HCl}^{slow}(t)\right]=\frac{Signal_{HCl- A1}^{sens}}{sens_{HCl- A1}} $$

(4) A comparable slow SO_2_ time series, [SO_2_^slow^(*t*)], is simulated by sensor response modelling by applying the time response properties of SRM5 to [SO_2_^inv^(t)], Eqs. 24–26 (note that SRM5 can alternatively take [SO_2_] from standard analysis as input given *T*_90_ for HCl-A1 > > *T*_90_ for SO2-AE).

24 $$ \left[{SO_2}^{slowA}(t)\right]=\left[{SO_2}^{slowA}\left( t-1\right)\right]\cdot {F}_{HCl- A1}^{sensA}+\left[{SO_2}^{inv}(t)\right]\cdot \left(1-{F}_{HCl- A1}^{sensA}\right) $$

25 $$ \left[{SO_2}^{slowB}(t)\right]=\left[{SO_2}^{slowB}\left( t-1\right)\right]\cdot {F}_{HCl- A1}^{sensB}+\left[{SO_2}^{inv}(t)\right]\cdot \left(1-{F}_{HCl- A1}^{sensB}\right) $$

26 $$ \left[{SO}_2^{slow}(t)\right]={W}_{HCl- A1}^{sens}\cdot \left[{SO_2}^{slow B}(t)\right]+\left(1-{W}_{HCl- A1}^{sens}\right)\cdot \left[{SO_2}^{slow B}(t)\right] $$

(5) Finally, a scatter plot of [HCl^slow^(*t*)] vs [SO_2_^slow^(*t*)] with linear regression is used to determine the gas ratio *R*_HCl/SO2_. Further illustration is given in Fig. 8b.

### Analysis of H_2_S/SO_2_ in Mt. Etna plume

Multi-Gas H_2_S detection at Mt. Etna is extremely challenging due to the H_2_S poor emissions and strong SO_2_ interference on the measurement. H_2_S/SO_2_ from Multi-Gas has only previously been reported at Mt. Etna using a specific H_2_S sensor setup with filter scrubber for SO_2_ (Aiuppa et al. 2011; Shinohara et al. 2011). Using the SRM analysis outlined above, we simulate the H_2_S-AE sensor signal and compare to the measured H2S-AE signal to evaluate a best estimate of plume H_2_S/SO_2_.

**Fig. 9:**
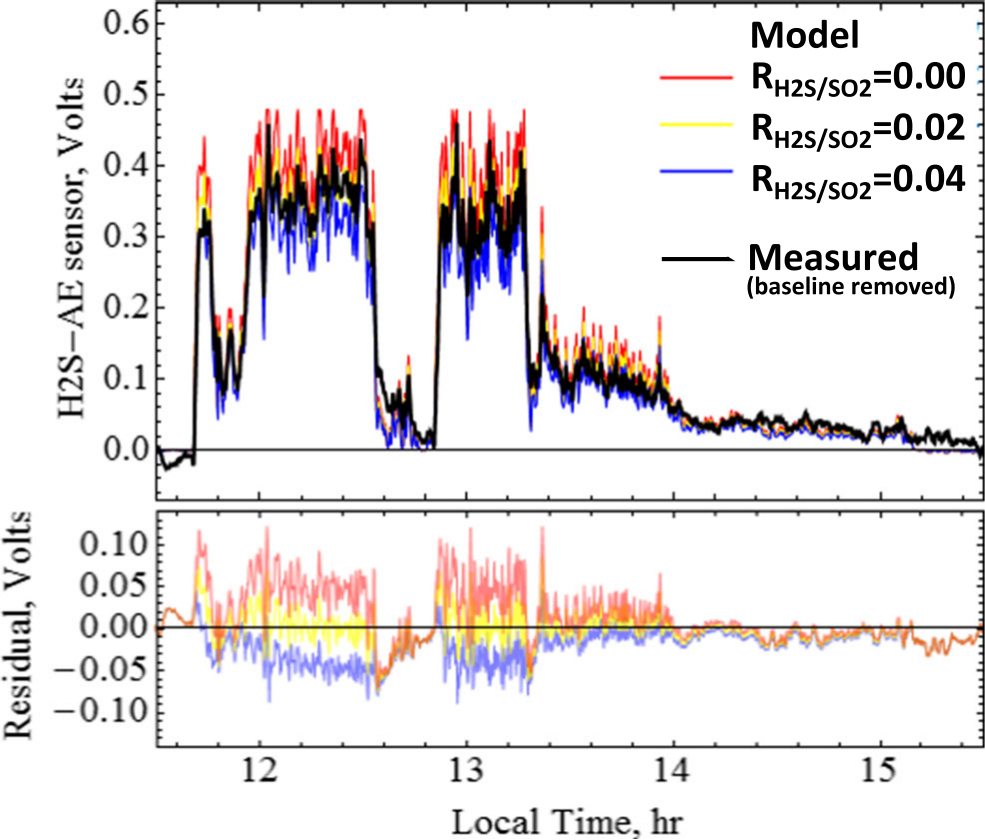
Analysis of H_2_S/SO_2_ using the SRM approach of Fig. 8. The measured H_2_S-AE signal is compared to simulated H2S-AE signals that assume three specified H_2_S/SO_2_ gas ratios. Best agreement is found for H_2_S/SO_2_ = 0.02, as shown by the residual (simulated-measured)

The simulated and observed H_2_S sensor signals are shown in Fig. 9, for three specified H_2_S/SO_2_ molar gas ratios. Best agreement is found for H_2_S/SO_2_ = 0.02, with clear under- and over-estimation for H_2_S/SO_2_ = 0.00 and 0.04, respectively. Thus, we estimate H_2_S/SO_2_ = 0.02 (0.01–0.03) for Mt. Etna (range robust to a 5% variability in (cross)-sensitivities, see Fig. S3). No clear differences could be detected between VOR, NEC and BN emissions. This H_2_S/SO_2_ ratio is quantitatively consistent with previously reported H_2_S/SO_2_ from filter-pack, diffusion tubes and the previous specific (interference-free) Multi-Gas sensor, Table 2. In comparison, standard analysis yields large scatter in H_2_S vs SO_2_, Fig. S4, even though the presence of H_2_S is evident during periods of sustained gas exposure (e.g. ~0.6 ppmv, alongside ~32 ppmv SO_2_, Fig. 6, i.e. H_2_S/SO_2_~0.02). Whilst averaging can improve signal-to-noise on standard analysis, the SRM approach is more robust to biases, particularly under episodic plume exposure. A higher data sampling rate is recommended to improve noise in future SRM analysis.

**Table 2:** Molar H_2_S/SO_2_ ratios (and range) at Mt. Etna reported from this and previous studies

	H_2_S/SO_2_ molar ratio	Method
This study	0.02 (0.01–0.03)	Multi-Gas
Aiuppa et al. (2011)	0.007 (0.0046–0.01)	Multi-Gas
Aiuppa et al. (2007b)	0.02	Diffusion tube (bulk plume)
	0.01	Filter pack at VOR
	0.05	Filter pack at NEC
	0.02	Bulk plume (1:1 VOR:NEC)
Aiuppa et al. (2005a)	0.05 (0.04–0.07)	Filter pack

### Analysis of HCl/SO_2_ in Mt. Etna crater’s emissions

Our detection of Mt. Etna plume HCl by electrochemical sensor builds on the prototype of Roberts et al. (2012). Here, the improved HCl electrochemical sensor (HCl-A1) exhibits a more stable sensor baseline (Fig. 6) achieved primarily by a change of composition and design of the working electrode (Alphasense, pers. com.) and has been more comprehensively characterized in terms of cross-sensitivities and response times (“Sensor characterization: sensitivity, cross-sensitivities, T90 and SRM”).

**Fig. 10:**
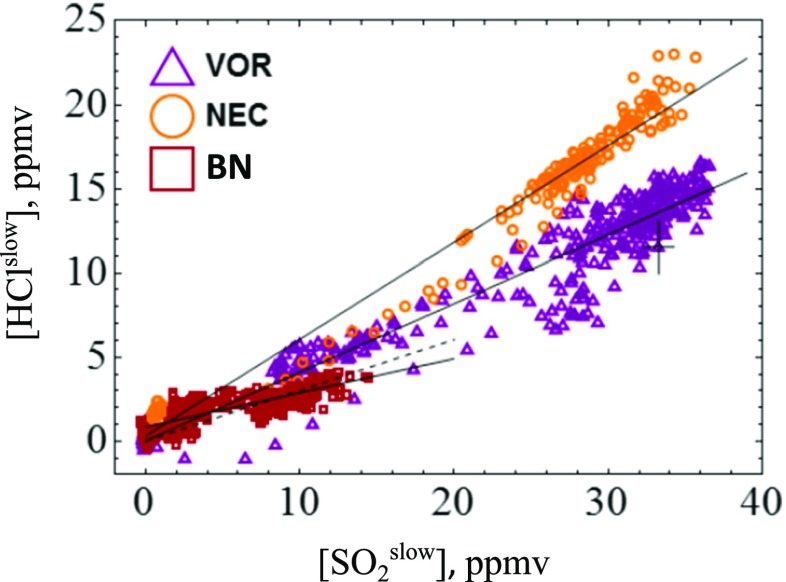
**a** Analysis of HCl/SO_2_ using the SRM approach of Fig. 8. Scatter plot of [HCl^slow^] and [SO_2_^slow^] gas abundances with linear regressions (*black lines*) for each crater emission yields HCl/SO_2_ ratios of 0.41 (0.38–0.43), 0.56 (0.54–0.60) and 0.20 (0.17–0.33) for VOR, NEC and BN, respectively. Also shown is the standard deviation in the data and, for BN, a linear regression forced through zero (*dotted line*)

The SRM analysis approach outlined in “Development of SRM data analysis approach for H_2_S/SO_2_ and HCl/SO_2_ gas ratios”, Fig. 8, was used to convert the sensor signals into slow response [HCl^slow^] and [SO_2_^slow^] outputs that can be directly compared in a scatter plot, Fig. 10. This used a molar H_2_S/SO_2_ ratio of *R*_H2S/SO2_ = 0.02 (range 0.01–0.03) and H2S-A1 cross-sensitivity to H_2_S of 210% (range 170–250%), following “Sensor characterization: sensitivity, cross-sensitivities, T90 and SRM” and “Analysis of H_2_S/SO_2_ in Mt. Etna plume”.

Figure 10 shows distinct HCl/SO_2_ for VOR, NEC and BN. [HCl^slow^] and [SO_2_^slow^] are well-correlated (cf standard analysis, Fig. S5), finding *R* ^2^ = 0.92, 0.98 and 0.69 at the respective craters with linear regression used to determine gas ratios. For VOR and NEC, the analysis yields HCl/SO_2_ of 0.41 (0.38–0.43) and 0.58 (0.54–0.60), respectively (range reflects possible H_2_S/SO_2_ of 0.01–0.03 and cross-sensitivity of 170–250%). These can be correspondingly written as SO_2_/HCl molar ratios of 2.45 (2.33–2.63) and 1.72 (1.66–1.85) for VOR and NEC, respectively. The (more dilute) BN plume exhibited much poorer correlation in [HCl^slow^] and [SO_2_^slow^]. BN HCl/SO_2_ is thus more uncertain, but estimated as 0.20 (0.17–0.33), i.e. SO_2_/HCl of 5.0 (3.0–5.8). Time series of [SO_2_] and [HCl] (standard analysis), [SO_2_^slow^], [HCl^slow^] (and with interference), Figs. S6–S8, illustrate how the slower rise in HCl upon plume exposure is more closely reproduced by SO_2_^slow^ than SO_2_ (standard analysis). Response over longer timescales than simulated here (our SRM-analysis is based on 10 min calibrations, see “Sensor characterization: sensitivity, cross-sensitivities, T90 and SRM”) might additionally contribute to the observed rising HCl signal (Fig. 6) and should be investigated for more prolonged plume exposures.

The Multi-Gas SO_2_/HCl is within the ranges reported from filter packs and remote-sensing FTIR, Table S1. Our lower SO_2_/HCl found at NEC than VOR agrees with filter-pack sampling by Aiuppa et al. (2005a) over 2004 who reported mean estimates of 1.32 and 2.99 mol mol^−1^ at these craters, respectively. Furthermore, the molar ratios are in very good quantitative agreement with recent 2010–2012 time-averaged sampling at Mt. Etna by Wittmer et al. (2014), Fig. 11. This agreement supports our Multi-Gas HCl measurement of distinct Cl/S ratios at NEC and central (VOR, BN) emissions (with weaker differences apparent between VOR and BN).

**Fig. 11:**
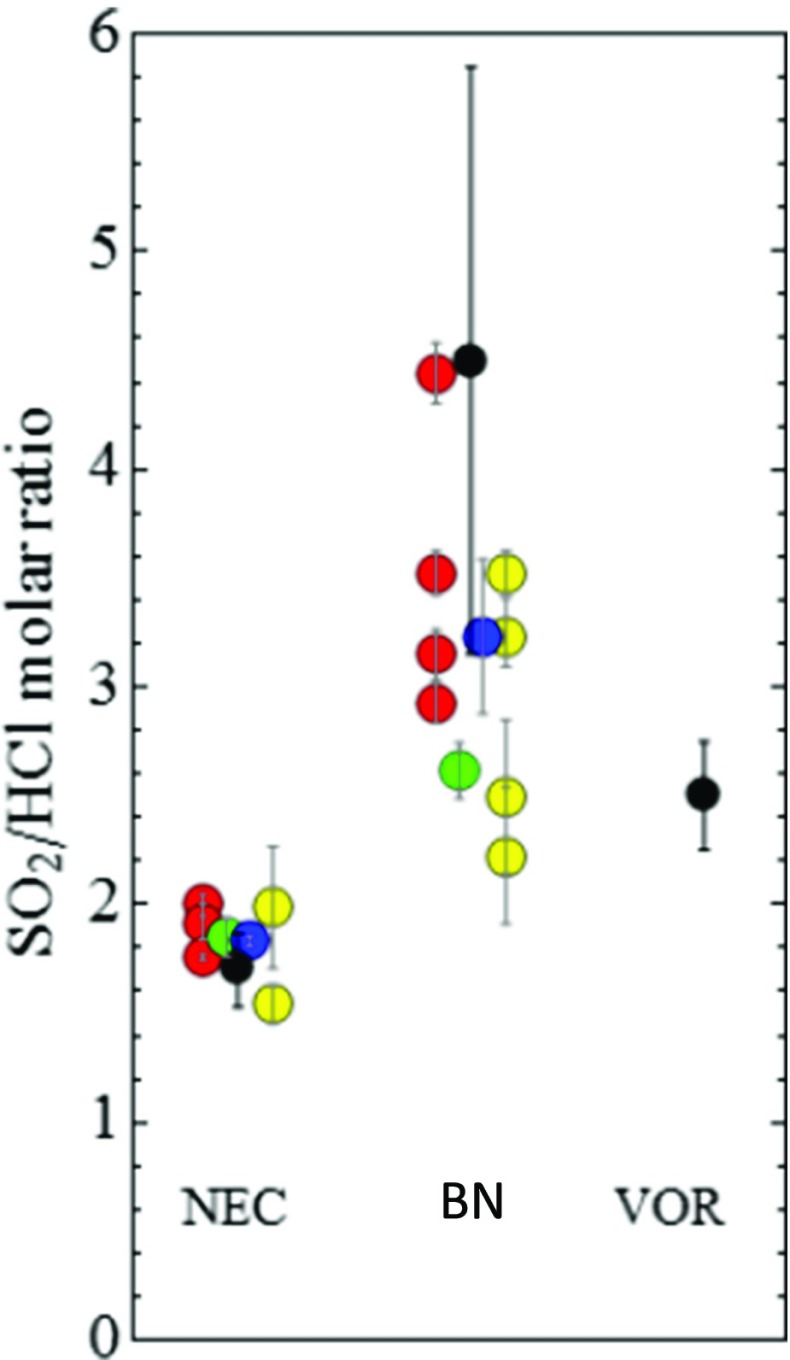
Comparison of SO_2_/HCl molar ratios recently reported at Mt. Etna summit craters. Gas ratios by Multi-Gas (this study, *black*) are compared to those reported by Wittmer et al. (2014) from time-averaged sampling over 2010–2012, using Dreshel bottle (*red*), Small Raschig-Tube (*green*), Big Raschig-Tube (*blue*) and Filter-packs (*yellow*)

## Discussion

This study demonstrates Multi-Gas H_2_S/SO_2_ and HCl/SO_2_ analysed by new SRM approaches. In particular, SRM improves accuracy of H_2_S/SO_2_ from standard Multi-Gas sensors for H_2_S-poor plumes. The determined H_2_S/SO_2_ molar ratio, 0.02 (0.01–0.03), can be used to estimate the temperature at which the H_2_S-SO_2_ magmatic gas equilibrium, R1, is quenched at Mt. Etna. Our calculation follows Aiuppa et al. (2011) where the ratio of SO_2_ and H_2_S fugacities, f*SO*_*2*_/f*H*_*2*_ *S*, can be replaced by the reciprocal of our measured [H_2_S]/[SO_2_] molar ratio. This is related to the oxygen fugacity, fO_2_, H_2_O fugacity, fH_2_O, and the highly positively temperature-dependent equilibrium constant, *K*_T_, by Eq. 27.

R1 $$ {H}_2{S}_{(g)}+\frac{3}{2}{O}_{2(g)}\leftrightarrow { S O}_{2(g)}+{H}_2{O}_{(g)} $$

27 $$ \log \frac{fSO_{2(g)}}{fH_2{S}_{(g)}}= \log {K}_T+\left(\frac{3}{2} \log {fO}_{2(g)}\right)- \log {fH}_2{O}_{(g)} $$

Our Mt. Etna SO_2_-H_2_S-HCl observations combined with CO_2_-H_2_O (see Supplementary material, Fig. S9) indicate a magmatic gas emission with around 90–95% H_2_O (by volume). Assuming a H_2_O content of 80% (to account for possible presence of unmeasured species such as HF, and noting low dependence of Eq. 27 on fH_2_O in any case), i.e. fH_2_O of 0.8 (1 bar) pressure, rearrangement of Eq. 27 can provide the temperature if the oxygen fugacity is known. Using the petrological estimate of Mt. Etna fO_2_ which is at Ni-NiO buffer +0.35, (Métrich and Clocchiatti 1996), and H_2_S/SO_2_ = 0.02 yields a quenching temperature of 800–900 °C, slightly lower than the inferred temperature of magma emission, ~1100 °C (Métrich and Rutherford 1998). This finding is similar to that of Aiuppa et al. (2011), as expected, since the reported H_2_S/SO_2_ is similar. However, in both calculations, it is assumed that the gas redox state remains similar to its parental magma and that the magma redox state inferred for reservoir conditions is identical to that reached when erupted (see Burgisser and Scaillet 2007). Decompressing magma may get either more reduced or oxidized relative to reservoir conditions upon ascent, differences in fO_2_ reaching a log unit, which translates into a temperature difference of about 100–150 °C (for instance the same H_2_S/SO_2_ ratio of 0.02 implies a temperature of 1100 °C at NNO-0.7). Regardless of associated uncertainties, such a broad agreement in temperatures supports the notion that, to a first order, measured H_2_S/SO_2_ ratios by Multi-Gas correctly capture magmatic conditions, hence likely to give insight to deep seated processes. Whilst Aiuppa et al. (2011) used a specific Multi-Gas set-up with interference-free H_2_S sensor, our SRM-analysis improves accuracy of H_2_S/SO_2_ in H_2_S-poor volcanic plumes using standard Multi-Gas sensors (where H_2_S exhibits cross-sensitivity to SO_2_).

**Fig. 12:**
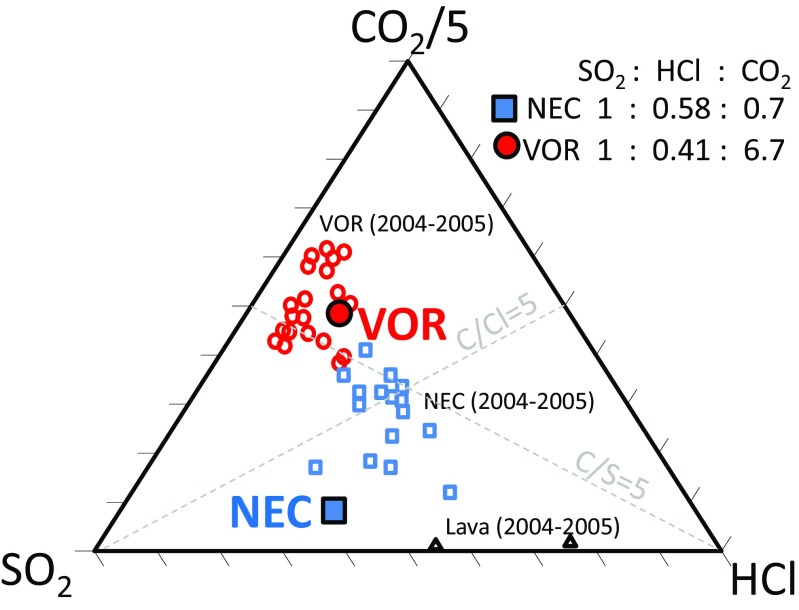
HCl-SO_2_-CO_2_ composition in this study at VOR and NEC shown on a *triangle plot* alongside composition reported by Aiuppa et al. (2006) during an effusive event. Plotting software from Graham and Midgley (2000)

SRM analysis of a newly characterized Multi-Gas HCl sensor yielded distinct HCl/SO_2_ at the three craters in good agreement to recent observations (Wittmer et al. 2014; Fig. 11). Our Multi-Gas observations provide a near-instantaneous overview of all three summit crater emissions, which seems rarely reported by other techniques, likely due to logistical reasons (as more time-consuming/power intensive). The HCl-SO_2_-CO_2_ data show general overlap in parameter space with reported compositions during and following an effusive event (Aiuppa et al. 2006; Fig. 12). The observed low SO_2_/HCl alongside low CO_2_/SO_2_ can be interpreted as resulting from a fractionated magma that is somewhat depleted in SO_2_ and CO_2_. Partial (fractional) gas depletion of magma has previously been suggested at Mt. Etna, e.g. Burton et al. (2003) following eruption events, or within the shallow conduit, e.g. Aiuppa et al. (2002, 2011). The greater SO_2_/HCl at VOR than NEC suggests either that the magma source for VOR is slightly less fractionated than NEC and/or that VOR gas comes from slightly deeper levels than NEC gas, noting the tendency for halogens to degas from melt and outgas from magma at lower pressures than SO_2_ or CO_2_, but that their subsurface transitions across different phases are complex and also depend on temperature and melt composition. Combining the Multi-Gas HCl/SO_2_ ratios (0.2–0.58 mol/mol) with Mt. Etna SO_2_ gas flux (1800–2100 t day^−1^ during the campaign), monitored by the INGV Ultraviolet scanning spectrometer FLAME network (Salerno et al. 2009), yields an HCl emission flux of several hundred tons per day. Whilst bulk plume HCl/SO_2_ has been measured at Mt. Etna since 2000 using FTIR in solar occultation mode (e.g. Burton et al. 2003), our new Multi-Gas sensing of HCl enables to characterize HCl emissions from individual craters, including during night.

The low cost of the HCl sensors (~100 euros) can facilitate wider application of the technology beyond Mt. Etna to other H_2_S-poor HCl-rich emissions such as Masaya (Nicaragua), Ambrym (Vanuatu) and Villarrica (Chile). Further tests are being undertaken for HCl sensing in more H_2_S-rich plumes that should use sensor-specific (rather than batch) cross-sensitivities to remove the H_2_S interference. Use of filters to remove H_2_S but not HCl will also be considered but is challenging. Integration of HCl sensors into permanent Multi-Gas installations for continuous emissions monitoring would be of interest to trace in real-time changes in Cl/S that can be associated with changing volcanic activity (varying by order-of-magnitude, see “Introduction”). However, the feasibility of continuous and long-term HCl monitoring by Multi-Gas requires further in-field tests of HCl-A1 sensor performance, stability and response. There is furthermore a general need to test the (wide-spread) use of laboratory sensor characterisations in the analysis of Multi-Gas field measurements at volcanoes.

## Conclusions

Measurements of the composition of volcanic emissions help researchers monitor and predict hazardous volcanic eruptions and assess downwind plume impacts. This study introduces real-time in situ HCl detection by Multi-Gas electrochemical sensors, with improvements made to analytical accuracy of Multi-Gas-measured H_2_S/SO_2_ and HCl/SO_2_ gas ratios by modelling the sensor response. The techniques are demonstrated in a field campaign at Mt. Etna on 2 October 2013 when two Multi-Gas instruments operating at 0.1–0.5 Hz were co-deployed to consecutively sample emissions from the three summit craters, Voragine (VOR), North-East Crater (NEC) and Bocca Nuova (BN), respectively.

A new Multi-Gas instrument, Multi-Gas^Direct^, contains electrochemical sensors for HCl, SO_2_ and H_2_S, which were directly exposed to the atmosphere. This removes the need for a pump, enabling a lighter and lower power instrument (hence easier in-field deployment with longer battery lifetime). The Multi-Gas SO_2_ sensor has negligible interferences, but laboratory calibrations show that the H_2_S sensor has a 14 ± 0.5% cross-sensitivity to SO_2_ and the HCl sensor has a 170–250% cross-sensitivity to H_2_S. The HCl sensor also exhibits a ~50% cross-sensitivity to HBr, but this can be neglected given HCl > > HBr in the volcanic emission. Laboratory characterization of the sensor response times found T90 = 12 s for SO2-AE, T90 = 20–50 s for H2S-AE and T90 = 100–250 s for HCl-A1. The combined effects of sensor response times, sensitivities and cross-sensitivities in the Multi-Gas field-data were deconvolved by signal processing algorithms to yield HCl/SO_2_ and H_2_S/SO_2_ molar gas ratios, finding H_2_S/SO_2_ = 0.02 (0.01–0.03) and HCl/SO_2_ = 0.44 (0.43–0.45), 0.61 (0.60–0.61) and 0.29 (0.23–0.34) for VOR, NEC and BN, respectively. These gas ratios agree with recent time-averaged sampling (Wittmer et al. 2014), confirming persistent differences in the crater HCl emissions. A second Multi-Gas instrument, Multi-Gas^Pump^, of traditional pumped design containing SO_2_, CO_2_ and H_2_O sensors was co-deployed, enabling cross-comparison of the SO_2_ measurement. The observed SO_2_-HCl-H_2_S-CO_2_-H_2_O compositions across the three craters reflect Mt. Etna outgassing processes. The H_2_S/SO_2_ indicates quenching at 800–900 °C, and we infer the presence of a partially evolved magma from SO_2_/HCl and CO_2_/SO_2_.

This study demonstrates Multi-Gas sensing of HCl emissions at Mt. Etna craters. The low-cost (~100 euros) of the HCl sensors can facilitate application to H_2_S-poor, HCl-rich volcanic plumes elsewhere, e.g. Masaya (Nicaragua). Future work will evaluate sensor performance in H_2_S-rich plumes and over longer timescales. We emphasize that accurate determination of Multi-Gas gas ratios requires inclusion of the effects of differing sensor response times when data is analysed (achieved here using Sensor Response Modelling). We encourage further application of signal processing/systems engineering in this area and also emphasize the need for field validation of laboratory-derived Multi-Gas sensor properties.

## Electronic supplementary material

ESM 1 (DOCX 1653 kb)
