# Supplementary material for: Validation of a novel Multi-Gas sensor for volcanic HCl alongside H2S and SO2 at Mt. Etna
Source: Bull Volcanol. 2017 Apr 17;79(5):36. doi: 10.1007/s00445-017-1114-z (PMC6979509; doi:10.1007/s00445-017-1114-z)
Supplement: Supplementary file 1 — (DOCX 1653 kb) [file 445_2017_1114_MOESM1_ESM.docx]

**Supplementary Material**

**1. Testing of interferences on the HCl-A1 sensor**


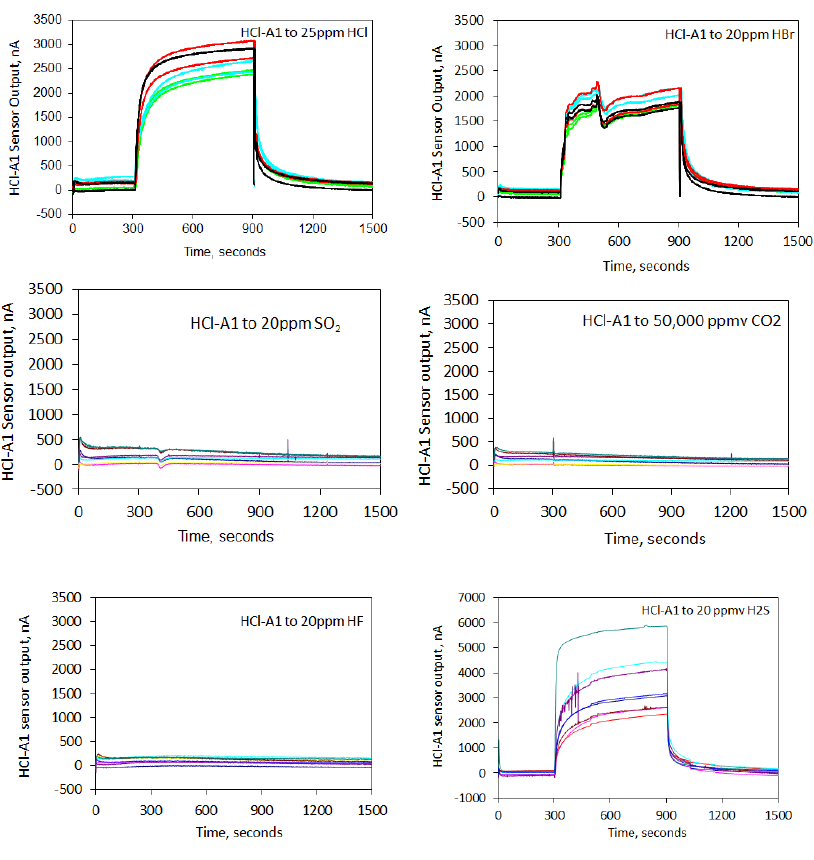


Figure S1. Laboratory tests of HCl-A1 sensors response to step pulses of gases: sensitivity to HCl, and cross-sensitivities to HBr, SO_2_, CO_2_, HF and H_2_S. Experiments were performed on batches of sensors. Gas exposure occurred between 300 and 900 seconds. There is no evidence of interferences from SO_2_, CO_2_ and HF. Interferences are evident for HBr and H_2_S, but only the H_2_S interference is significant under typical volcanic plume conditions (HBr << HCl). Percentage cross-sensitivity of H_2_S on HCl-A1 (170-250%, mean 210%) was derived by dividing the ratio of the maximum interference signal (nA) to the H_2_S calibration gas (ppmv) by the ratio of the sensitivity (nA) to the HCl calibration gas (ppmv) and multiplying by 100.

**2. HCl-A1 sensor repeated and long-term response to H_2_S and HCl**


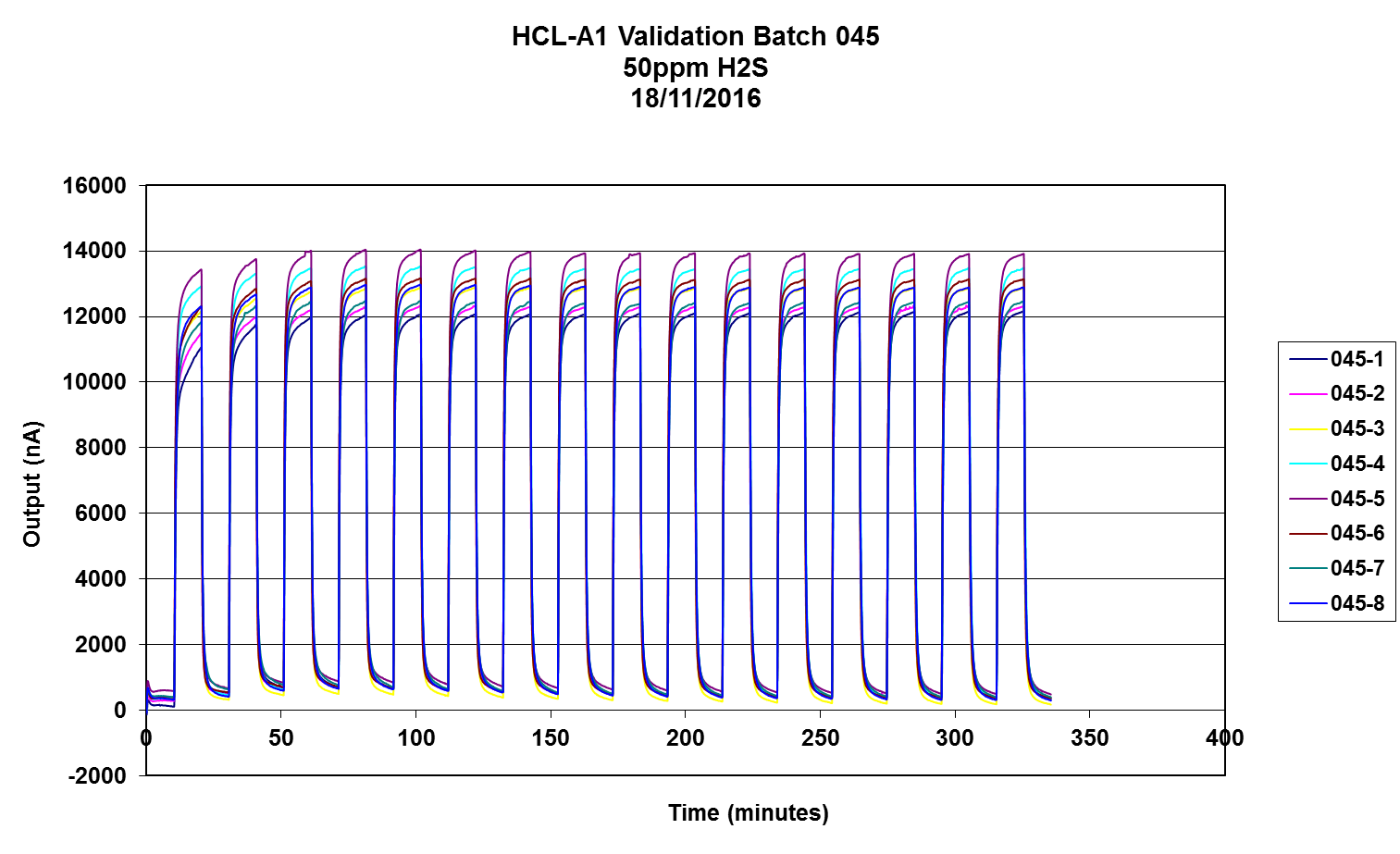


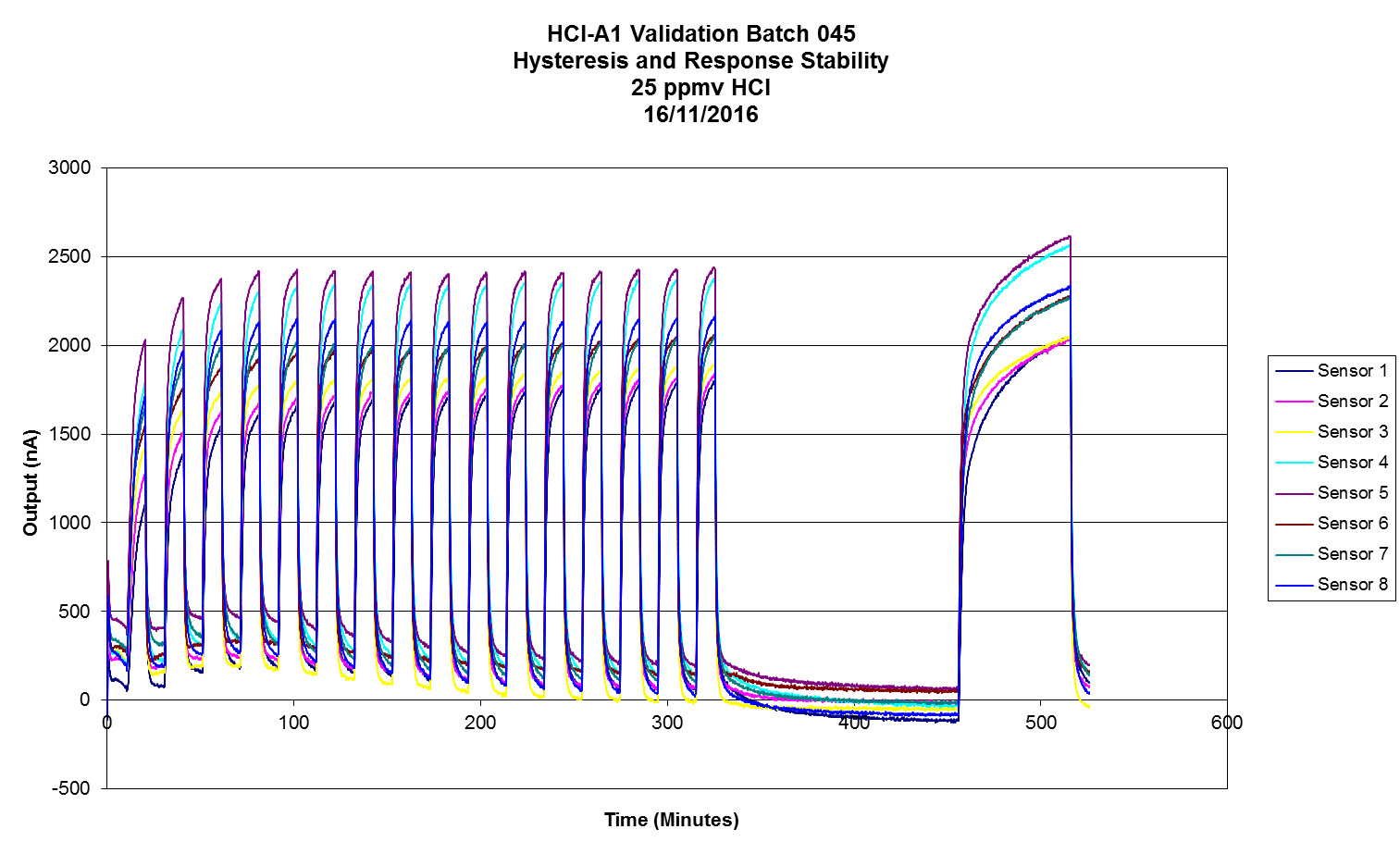


Figure S2 HCl-A1 repeated 10 min exposure tests to H_2_S and HCl show variability of 10% (2σ) sensitivity and 2 ppmv (2σ) baseline following initial exposure (first 3 cycles considered as warm-up period). Also shown is a long-duration 1 hr exposure of HCl-A1 signal to HCl.

**3. Uncertainty in H_2_S/SO_2_ by SRM analysis**


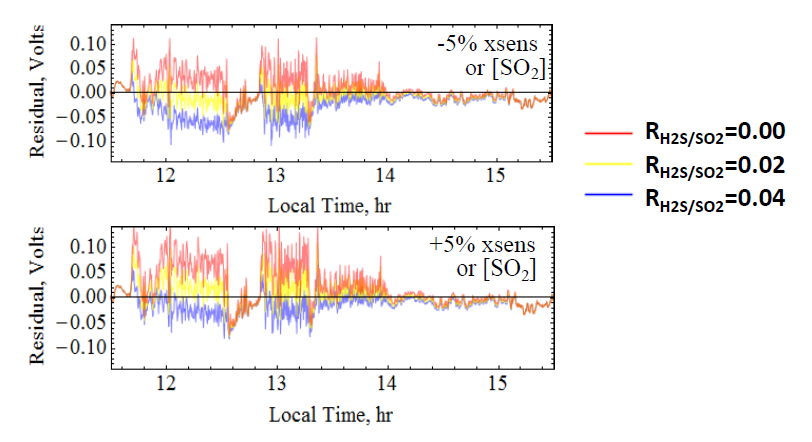


Figure S3. Uncertainties in analysis of H_2_S/SO_2_ using the SRM-approach. The measured H_2_S-AE signal is compared to simulated H2S-AE signals that assume three specified H_2_S/SO_2_ gas ratios. The residual (simulated-measured) found best agreement for H_2_S/SO_2_ = 0.02 (Figure 9). Here, residuals are calculated for cases with 5% higher/lower [SO_2_] or cross-sensitivity, xsens_SO2_, confirm the finding H_2_S/SO_2_ = 0.02 ±0.01.

**4. H_2_S/SO_2_ by standard analysis**


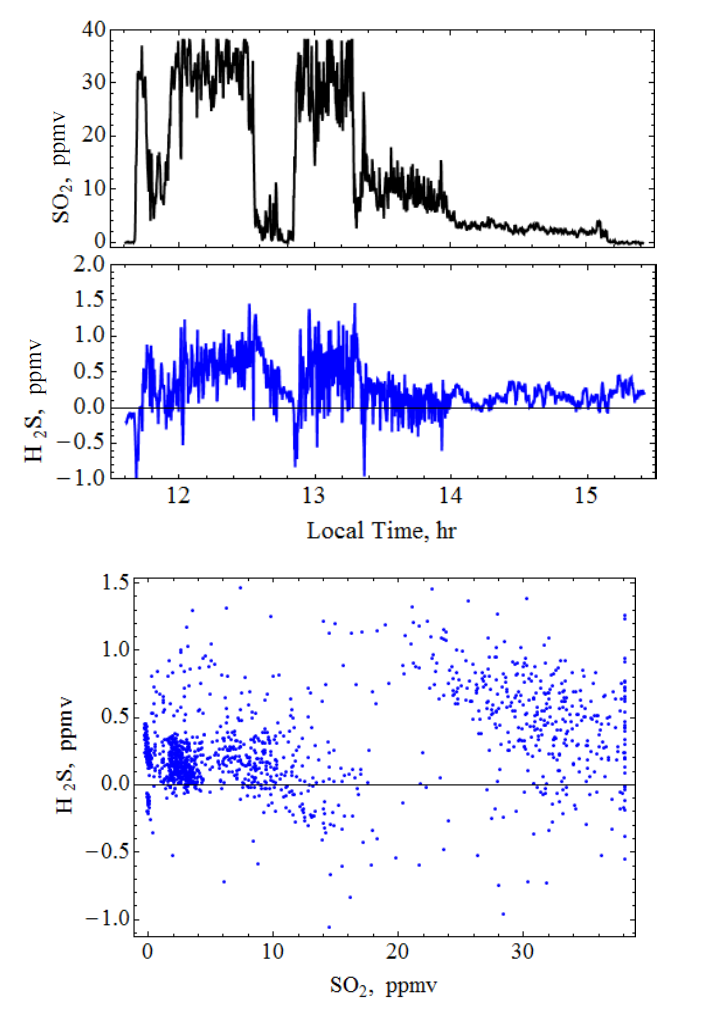


Figure S4. Scatter plot of SO_2_ and H_2_S obtained by standard analysis of the Multi-Gas data (E1-E2). High variability is caused by the differing sensor response times, and magnified by the removal of an interference (SO_2_ on H2S-AE), as described in Introduction Section 1.3.

**5. Multi-Gas^Direct^ measurement of HCl by standard and SRM-analysis**


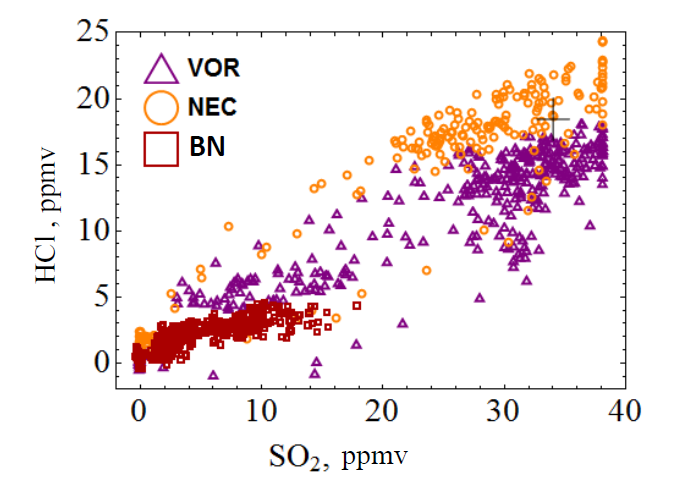


Figure S5. Scatter plot of HCl versus SO_2_ analysed by standard analysis identifies distinct HCl/SO_2_ gas ratios for the three craters but exhibits more variability than SRM-analysis Figure 10 ([HCl^slow^] versus [SO_2_^slow^]. Greater variability (and possible bias in HCl/SO_2_) in standard analysis is expected for cases where plume exposure to the sensors is more episodic.


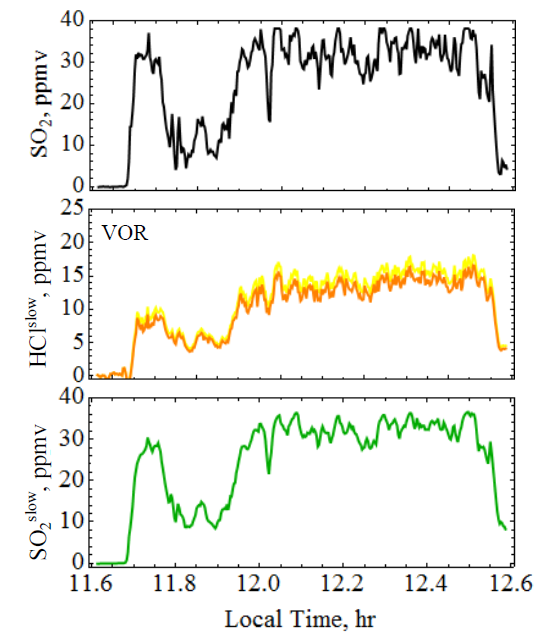


Figure S6. Multi-Gas^Direct^ measurements at VOR: SO_2_ by standard analysis, HCl^slow^ and SO_2_^slow^ by SRM analysis. Also shown (yellow) is HCl^slow^ + H_2_S interference.


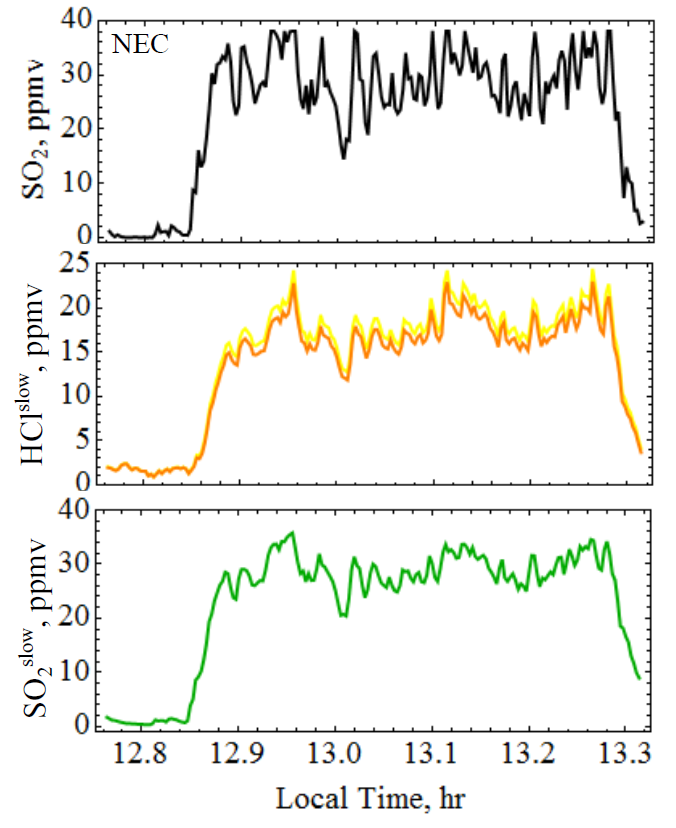


Figure S7. Multi-Gas^Direct^ measurements at NEC: SO_2_ by standard analysis, HCl^slow^ and SO_2_^slow^ by SRM analysis. Also shown (yellow) is HCl^slow^ + H_2_S interference.


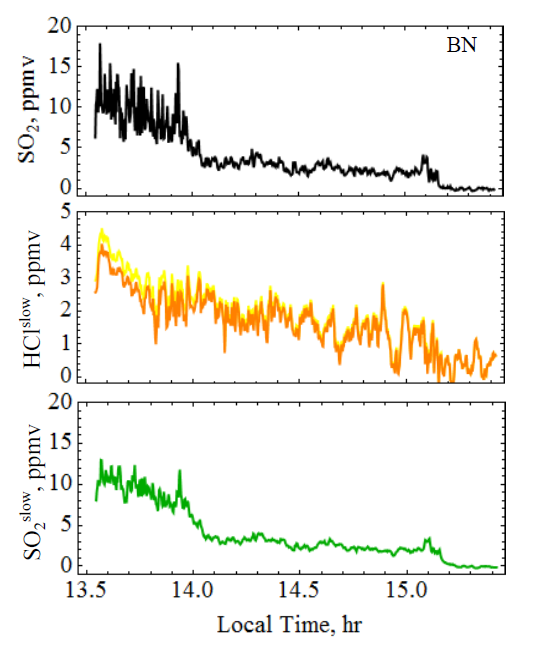


Figure S8. Multi-Gas^Direct^ measurements at BN: SO_2_ by standard analysis, HCl^slow^ and SO_2_^slow^ by SRM analysis. Also shown (yellow) is HCl^slow^ + H_2_S interference.

Table S1. Molar SO_2_/HCl ratios reported in Mt Etna bulk plume, and specific cases with near-simultaneous measurements at 2-3 craters. Standard error or gas ratio range also shown. TAS = Time-Averaged Sampling. FP = Filter-pack, SRT = Small Raschig-Tube, BRT = Big Raschig-Tube, DB = Dreschel bottle, FTIR = Fourier Transform Infra-Red Spectroscopy, MI: melt inclusions. *uncertain FP data: likely saturation in S can yield anomalously low SO_2_/HCl according to Wittmer et al. (2014).

|  | Method | Date (dd.mm.yyyy) | Bulk Plume | NEC | VOR | BN | Other |
| --- | --- | --- | --- | --- | --- | --- | --- |
| This study | Multi-Gas | 2013.10.02 | - | 1.7 (1.6-1.9) | 2.5 (2.3-2.7) | 4.5 (3.0-5.8) | - |
| Wittmer et al. (2014) | DB  SRT  BRT  FP | 2010 – 2012  2010 – 2012  2010 – 2012  2010 – 2012 | -  -  -  - | 1.89 ± 0.11  1.85 ± 0.08  1.83 ± 0.08  1.79 ± 0.18 | -  -  -  - | 3.5 ± 0.7  2.6 ± 0.1  3.2 ± 0.2  3.1 ± 0.9 | -  -  -  - |
| Voigt et al. (2014) | Aircraft (1-4 hr downwind) &FP | 2011.09.29-30 | 4.4 (layers: 5.4, 3.5) | - | - | 3.1 ± 0.1 | - |
| Unpublished INGV data  Aiuppa pers. com. | FP  FP | 2010.06.24  2011.06.24 | - | 1.56  1.89 (1.79-1.99) | - | 2.63  3.05 (2.88-3.21) | - |
| La Spina et al. (2010) | FTIR with custom IR source | 2008.07.21  2009.09.03  2009.08.31 | - | 2.9 ± 0.116  2.8 ± 0.11  7.7 ± 0.23 | - | 2.3 ± 0.069  5.5 ± 0.165  2.9 ± 0.12 | - |
| Martin et al. (2008) | FP (possibly saturated*) | 2005.08-09 |  | 0.86* (0.5-1.2) | 2.2* (1.77-2.9) |  | - |
| Pyle and Mather (2009)  Aiuppa et al. (2005) | FP annual geometric mean  FP mean | 2004  2004 |  | 1.16  1.32 | 2.99  2.99 |  | - |
| Aiuppa (2009) | Review: FP & other studies | FP: 2003 - 2007 & earlier | 2.4 (0.1 – 14.7) | 0.5 – 3 (typical) | (see CC) | (see CC) | 0.8 – 9 (typical CC) |
| Aiuppa et al. (2007) | Diffusion Tubes | 2005.07-08  2004.07-08  2003.10-11  2002.05-06 | 2.2 ± 0.5  1.7 ±1.0  1.0 ± 0.2  4.9 ± 0.9 | - | - | - | - |
| Aiuppa et al. (2004a) | FP | 2002.10-2003.01 | - | - | - | - | 0.1-6.8 (eruptive) |
| Aiuppa et al. (2004b) | Diffusion Tubes | 2002.12-2003.02 | - | - | - | - | 2-8 (eruptive) |
| Burton et al. (2003) | Sun aligned FTIR | 2001.05  2001.07 | 2.91  3.22 | 2.91 |  |  | 0.84 (fissure) |
| Aiuppa et al. (2002) | FP | 2001.05-07  2001.07 | - | - | - | 1.1 – 2.6 | -  0.1-0.9 (fissure) |
| Caltabiano et al. (2004) | Sun aligned FTIR | 2000.03-12 | 1.5-6.5 | - | - | - | - |
| Allard et al. (2005) | FTIR | 2000.05-06 | 2.5 |  |  |  | 10 (SEC fountain)  1.8 (lava) |
| Francis et al. (1998) | Sun aligned FTIR | 1997.06.14  1997.10.26 | 4.0  4.6 | - | - | - | - |
| Francis et al. (1995) | Sun aligned FTIR | 1994.09.22  1994.09.25 | 3.29 ± 0.33  4.13 ± 1.62 | - | - | - | - |
| Spilliaert et al. (2006b) | MI closed system degassing |  |  |  |  |  | 3.7-9.7 (predicted) |

**6. Co-deployment of a pumped Multi-Gas**

**6.1 Multi-Gas^Pump^ instrument description**

A second Multi-Gas, Multi-GAS^Pump^ was co-deployed using a traditional pumped instrument from INGV (similar to e.g. Aiuppa et al., 2007), whereby air is drawn over a suite of mini-sensors (for SO_2_, CO_2_), with measurements recorded at 0.5 Hz. Internal temperature of the Multi-GAS^pump^ is typically higher than ambient and was monitored alongside relative humidity (T-RH, Galltech-Mela) and pressure (Freescale MPX). The sensors included an electrochemical SO_2_ sensor (manufactured by City Technology, version 3ST/F, range 0-200 ppmv, repeatability 1%) an infra-red spectroscopic sensor for CO_2_ (Gascard NGA – Edinburgh Instruments, temperature and pressure corrected; range 0–3000 ppmv, accuracy ±2%). Data are stored on a data-logger, specifically designed by INGV-Palermo, able to sample at a frequency of 0.5 Hz and at electronic resolution equivalent to 0.1 ppmv (CO_2_) or 0.01 ppmv (SO_2_). The infra-red CO_2_ sensor uses beer-lambert law to detect CO_2_ gas concentration which the Gascard electronics processing automatically converts to a 4-20 mA output proportional to ppmv gas abundance. This automatic conversion includes a temperature correction as well as a pressure correction to account for lower ambient pressure (P_actual_ ~ 700 hPa) at Mt Etna summit compared to calibration (P_calib_ = 1013 hPa).

The H_2_O measurement was determined from the RH sensor as a function of P, T and RH following the Buck equation (Buck, 1981), ES1, where *T* is the temperature (Celsius), *RH* the relative humidity (percent), and *P* the pressure (mb):

ES1 $[H_{2}O_{(g)}]=6.1121\cdot\left(1.0007+3.46\cdot10^{-6}\cdot P\right)\cdot Exp\left[\frac{17.502\cdot T}{240.97+T}\right]\cdot10^{4}\cdot RH\cdot P^{-1}$

**6.2 Multi-Gas^Pump^ measurements of CO_2_/SO_2_ and H_2_O/SO_2_**

To complete the Multi-Gas determination of major volcanic gases, CO_2_/SO_2_ and H_2_O/SO_2_ molar ratios from Multi-Gas^Pump^ are shown in Figure S9 for VOR and NEC. Measured gas ratios were variable in the more dilute BN, and are known to be challenging due to the H_2_O and CO_2_-rich background atmosphere. CO_2_/SO_2_ molar ratios at VOR and NEC are identified as 6.8 and 0.7, respectively (with overall error around 20%). However, the background CO_2_ is rather high (~550 ppmv). This may be due to baseline drift of the instrument or reflect enhanced CO_2_ at Mt Etna due to diffusive degassing, noting elevated CO_2_ in published data from previous Multi-Gas studies, e.g. Shinohara et al. (2008). A second CO_2_/SO_2_ ratio of 4.13 is reported at 14:00 by a continuously installed INGV Multi-Gas (similar instrument to Multi-Gas^Pump^). H_2_O/SO_2_ molar gas ratios in VOR and NEC crater emissions are 143 and 19, respectively, with a H_2_O background of around 4000 ppmv. Both CO_2_/SO_2_ and H_2_O/SO_2_ scatter plots indicate some variability in measured H_2_O or CO_2_ for a given SO_2_ abundance. This might reflect non-identical sensor response times or variations in gas-aerosol partitioning, or background variability. Combining these ratios yields H_2_O/CO_2_ of 3.0 and 38 mol mol^-1^ at VOR and NEC, respectively. Thus, VOR was more enriched in CO_2_ and H_2_O relative to SO_2_ compared to NEC. The general composition (i.e. H_2_O > CO_2_ > SO_2_) and magnitude of these gas ratios is consistent with previous Mt Etna studies. For example, Shinohara et al. (2008) report CO_2_/SO_2_ and H_2_O/CO_2_ ratios ranging from 0.6 to 30 and from 1 to 50 mol mol^-1^, respectively.


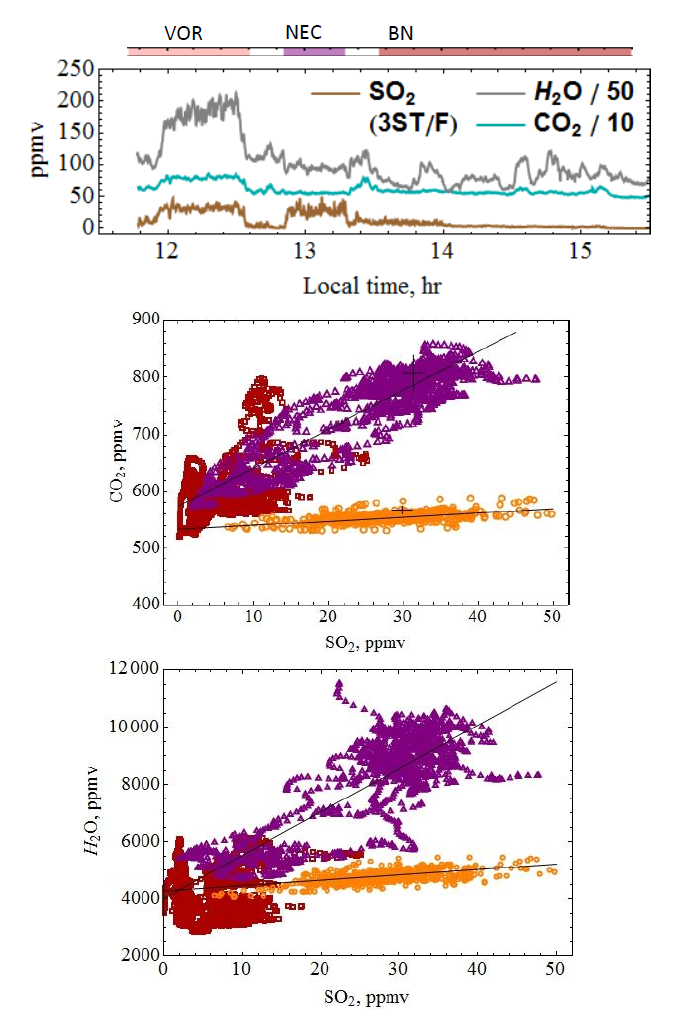


Figure S9. Time-series of SO_2_, CO_2_ and H_2_O from Multi-Gas^Pumped^ and scatter plots of CO_2_/SO_2_ and H_2_O/SO_2_.

**References**

Aiuppa AL, Federico C, Franco AN, Giudice G, Gurrieri S, Inguaggiato S, Liuzzo M, McGonigle AJ, Valenza M. Emission of bromine and iodine from Mount Etna volcano. Geochemistry, Geophysics, Geosystems. 2005 Aug 1;6(8).

Aiuppa, A., R. Moretti, C. Federico, G. Giudice, S. Gurrieri, M. Liuzzo, P. Papale, H. Shinohara, and M. Valenza (2007) Forecasting Etna eruption by real time evaluation of volcanic gas composition, Geology, **35**, 1115– 1118, doi:10.1130/G24149A.

Aiuppa A. (2009) Degassing of halogens from basaltic volcanism: Insights from volcanic gas observations, Chemical Geology, **263**, 99–109.

Aiuppa AL, Federico C, Giudice G, Gurrieri S, Paonita A, Valenza M., 2004a, Plume chemistry provides insights into mechanisms of sulfur and halogen degassing in basaltic volcanoes. Earth and Planetary Science Letters. **222**, **2**:469-83.

Aiuppa, A., S. Bellomo, W. D'Alessandro, C. Federico, M. Ferm, and M. Valenza (2004b), Volcanic plume monitoring at Mount Etna by diffusive (passive) sampling, J. Geophys. Res., **109**, D21308, doi:10.1029/2003JD004481.

Aiuppa A, Federico C, Paonita A, Pecoraino G, Valenza M. (2002) S, Cl and F degassing as an indicator of volcanic dynamics: the 2001 eruption of Mount Etna. Geophysical Research Letters, **29**, **11**, doi:10.1029/2002GL015032.

Allard P, Burton M, Muré F. (2005) Spectroscopic evidence for a lava fountain driven by previously accumulated magmatic gas, Nature, **433**, **7024**, 407-410, doi:10.1038/nature03246.

Burton M, Allard P, Mure F, Oppenheimer C.(2003) FTIR remote sensing of fractional magma degassing at Mount Etna, Sicily. Geological Society, London, Special Publications, **213**, **1**, 281-93.

Caltabiano T, Burton M, Giammanco S, Allard P, Bruno N, Murè F, Romano R. (2004) Volcanic gas emissions from the summit craters and flanks of Mt. Etna, 1987–2000. Mt. Etna: Volcano Laboratory (eds A. Bonaccorso, S. Calvari, M. Coltelli, C. Del Negro and S. Falsaperla), American Geophysical Union, Washington, D. C.. doi: 10.1029/143GM08.

Francis P, Maciejewski A, Oppenheimer C, Chaffin C, Caltabiano T. SO2 (1995), HCl ratios in the plumes from Mt. Etna and Vulcano determined by Fourier Transform Spectroscopy. Geophysical Research Letters, **22**, **13**, 1717-1720, 10.1029/95GL01657.

Francis P, Burton MR, Oppenheimer C. (1998) Remote measurements of volcanic gas compositions by solar occultation spectroscopy, Nature, 1998 , **396**, **6711**, 567-570, doi:10.1038/25115.

La Spina, A., Burton M., and Salerno G. G. (2010) Unravelling the processes controlling gas emissions from the central and northeast craters of Mt. Etna, Journal of Volcanology and Geothermal Research, 198, 3, 368-376.

Martin RS, Mather TA, Pyle DM, Power M, Allen AG, Aiuppa A, Horwell CJ, Ward EP (2008) Composition‐resolved size distributions of volcanic aerosols in the Mt. Etna plumes. Journal of Geophysical Research: Atmospheres, **113**, **D17**, doi: 10.1029/2007JD009648.

Pyle D. M. and Mather T. A. (2009), Halogens in igneous processes and their fluxes to the atmosphere and oceans from volcanic activity: A review, Chemical Geology, **263**, 110–121.

Shinohara, H., Aiuppa, A., Giudice, G., Gurrieri, S., and Liuzzo, M. (2008) Variation of H2O/CO2 and CO2/SO2 ratios of volcanic gases discharged by continuous degassing of Mount Etna volcano, Italy. Journal of Geophysical Research: Solid Earth, **113, B9**, doi:10.1029/2007JB005185.

Spilliaert, N., Allard, P., Métrich, N. and Sobolev, A.V. (2006a). Melt inclusion record of the conditions of ascent, degassing, and extrusion of volatile‐rich alkali basalt during the powerful 2002 flank eruption of Mount Etna (Italy). Journal of Geophysical Research: Solid Earth, **111**, **B4**, doi:10.1029/2005JB003934.

Spilliaert N, Métrich N, Allard P. (2006b) S–Cl–F degassing pattern of water-rich alkali basalt: modelling and relationship with eruption styles on Mount Etna volcano, Earth and Planetary Science Letters, **248**, **3**, 772-86, http://dx.doi.org/10.1016/j.epsl.2006.06.031.

Voigt, C., P. Jessberger, T. Jurkat, S. Kaufmann, R. Baumann, H. Schlager, N. Bobrowski, G. Giuffrida, and G. Salerno (2014). Evolution of CO_2_, SO_2_, HCl, and HNO3 in the volcanic plumes from Etna. Geophysical Research Letters, **41, 6**, 2196-2203.
